# Stability and change of spirituality following childbirth: longitudinal evidence from Swiss Household Panel data using multiple propensity score matching analyses

**DOI:** 10.3389/fpsyg.2026.1813504

**Published:** 2026-08-04

**Authors:** Friedemann Trutzenberg, Michael Eid

**Affiliations:** Division of Methods and Evaluation, Department of Education and Psychology, Freie Universität Berlin, Berlin, Germany

**Keywords:** causal inference, childbirth, life events, propensity score matching, spirituality, Swiss Household Panel, transition to parenthood

## Abstract

**Introduction:**

The birth of a child typically transcends parents’ daily lives and is frequently described as a spiritual experience. Yet, little is known about medium- or long-term effects of becoming parents on individuals’ spirituality. Spirituality is commonly conceptualised more broadly than religiosity and can refer to an orientation towards something divine, no matter whether this is a religious being or a non-religious entity. Its lifespan development and responsiveness to life events are not yet systematically understood, and existing evidence often does not allow for causal conclusions. Addressing this research gap, our study seeks to provide robust causal insights on the medium-term stability and change of spirituality following childbirth.

**Methods:**

Using longitudinal pre- and post-birth spirituality data from the Swiss Household Panel (SHP), we compared participants who had become parents for the first time between 2015 and 2018 with propensity score (PS)-matched childless and multiparous parent controls.

**Results:**

Across multiple robustness conditions regarding covariate selection, manifest and structural equation modelling analyses suggested a high average medium-term stability in spirituality (r ≥ 0.87; *ρ* ≥ 0.95) and no average spiritual change following childbirth among the first-time parents (*N* = 138) compared to the matched controls.

**Discussion:**

Given this high average stability, future research should focus on differential trajectories rather than average effects to better understand which individual and situational characteristics influence the development of spirituality following life events.

## 1 Introduction

The birth of a child transcends parents’ daily lives and is frequently described as a miracle or an existential or spiritual experience in qualitative interview studies across countries and religious backgrounds (e.g., Callister, 2004), resembling “sacred moments” (Pargament et al., 2025, p. 1).

However, it is not yet well understood whether these singular experiences are followed by lasting changes in parents’ spirituality.

Spirituality is often defined as individuals’ “search for the sacred” (Pargament, 1999, p. 12; see Davis et al., 2023; Pargament et al., 2013). Notably, *the sacred* does not solely refer to religious entities—it can also represent non-religious phenomena, e.g., connectedness with nature or the universe (e.g., Pargament et al., 2013). Accordingly, religiosity is seen as *one* of many ways to seek the sacred.

While, apart from medium-term fluctuations around the event, childbirth does not seem to cause more than slight long-term changes in well-being and personality, i.e., how individuals *are* and *feel* (e.g., Bühler et al., 2024; Krämer and Rodgers, 2020), there are reasons to assume that spirituality, i.e., how individuals *see life* and relate to “the sacred”, might be affected more strongly, especially by the birth of an individual’s first child: (1) Albeit being a fundamentally natural phenomenon, expectant nulliparous parents typically experience childbirth for the first time after having already spent decades on Earth. (2) Neither the course of delivery nor the newborn’s character can be planned. Against this background, childbirth may provide fertile ground for spiritual questions to arise: Have I really known life before? Is it really me who controls my life? Maybe there is something greater?

A systematic review conducted prior to the current study revealed that only a few non-retrospective longitudinal studies had investigated effects of life events on spirituality; most focused on different life stages and were difficult to compare (e.g., Read et al., 2014). Studies specifically addressing childbirth were cross-sectional and retrospective, used few items to measure spirituality, focused exclusively on mothers, and/or did not employ methods that allow for causal statements given biographies’ quasi-experimental nature (e.g., Berman et al., 2020; see Holland, 1986; see Lawes et al., 2025). Overall, the limited existing evidence suggests that childbirth may provoke long-term spiritual changes in some individuals, but results regarding the direction of the effect are ambiguous.

## 2 Materials and methods

To address the research gaps outlined above and to shed light on this important research question, the current study sought to provide precise, robust, and causal evidence on the medium-term stability and change in individuals’ spirituality following the birth of their first child. We used longitudinal, non-retrospective panel data collected before and after childbirth, compared first-time parents with *propensity score (PS)-*matched childless and multiparous parent controls, and performed numerous robustness checks.

To ensure reproducibility and replicability (see Davis et al., 2026; van Elk, 2019), comprehensive documentation of all methods and the analytical code are available at: https://doi.org/10.17605/OSF.IO/9N6G5.

### 2.1 Data^1^

Of more than 90 panel studies, we selected the *Swiss Household Panel* (SHP; SHP Group, 2022) for this study because it provides longitudinal data on spirituality from a representative sample (see ,Supplement 1). Besides, a Swiss sample appeared suitable for studying spirituality rather than institutional religiosity: Due to the increasing degree of secularisation in Switzerland (cf. Schweizer Radio und Fernsehen, 2024), spirituality may gain more importance in individuals’ lives than institutional religiosity (ibid.).

#### 2.1.1 Eligibility

A total of 48,596 individuals have lived in the SHP’s randomly selected private households in Switzerland during at least one of the first 22 annual survey waves (*W*; Voorpostel et al., 2022). All participants were eligible, except those with inconsistent entries on children, those with implausible interview dates (one individual), and women older than 66 years at baseline (the highest recorded age of a mother at birth in Switzerland; Neue Zürcher Zeitung, 2012).

#### 2.1.2 Ethics statement

SHP data were collected by the FORS centre (Swiss Centre of Expertise in the Social Sciences) which ensures full compliance with ethical guidelines. We used existing SHP data and had no direct contact with any participants.

### 2.2 Measures

#### 2.2.1 Spirituality

Unlike numerous basic questions repeated in each wave, the SHP *religion* module is administered every 3 years (Voorpostel et al., 2022). It contains seven items suitable for measuring spirituality in line with our conceptualisation (e.g., “Putting aside whether or not you would describe yourself as a religious person, how spiritual would you say you are?”; Stolz and Monnot, as cited in SHP Group, 2022, individual questionnaires W14, W17, W20. Reprinted with permission. ^©^ FORS – Swiss Centre of Expertise in the Social Sciences).

This item selection largely corresponds to the “interreligious” short form CRSi-7 of the *Centrality of Religiosity Scale* (Huber and Huber, 2012, p. 717). However, in line with the aforementioned conceptualisation, it contains only the six CRSi-7 items that capture non-institutional religious or non-religious forms of spirituality (partly due to reformulations, ibid.) to distinguish spirituality from institutional religiosity. The item cited above is not part of the CRSi-7.

The CRSi-7 has shown adequate to high internal consistency (0.67 < *α* < 0.84, 0.66 < *ω* < 0.85), time-invariant psychometric properties, and acceptable factorial validity in samples across the globe (e.g., Ackert, 2021).

We rescaled all seven items to a single scale ranging from 0 to 20. They are presented in Supplement 2.

#### 2.2.2 Childbirth events

We used two pieces of information to determine whether (and, if applicable, when) participants had become parents: (1) participants’ annually updated *number of children born* (i.e., not *children alive*) and (2) the birth months of participants’ children (who automatically become panel members; Voorpostel et al., 2022).^2^

#### 2.2.3 Matching variables

Numerous covariates were used during matching (see Supplement 3).

### 2.3 Design^3^

**Figure 1:**
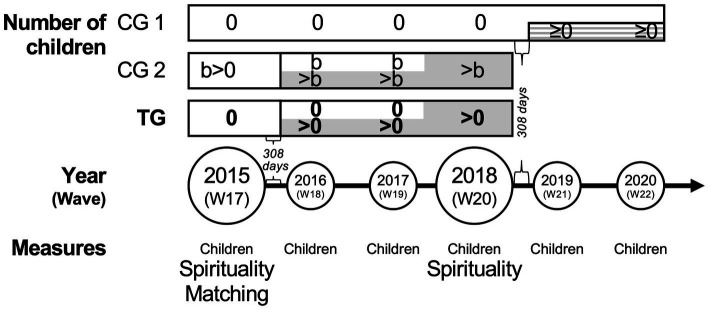
Londitudinal study design.

Figure 1 shows the longitudinal study design. We examined the SHP participants who had become parents for the first time between their W17 and W20 interviews (N = 138), applying a buffer of 308 days after the W17 interview, to ensure that participants were not pregnant. These were the only two SHP waves providing spirituality data for most participants at the time we conducted our analyses. Given the three-year measurement interval, our study was unable to capture short-term spiritual reactions and focused on medium-term stability and changes instead.

#### 2.3.1 Separate analyses by sex due to nested data

Owing to the household sampling design (Voorpostel et al., 2022), we identified 36 heterosexual couples among the first-time parents. This couple membership accounted for 76% (ICC = 0.76) of the variance in individuals’ baseline spirituality (manifest mean). Therefore, we conducted all analyses separately for individuals who identified as female or male on the SHP’s binary sex item (N_♀_ = 69).

#### 2.3.2 Double control group approach

To disentangle childbirth-induced changes from age-related or *missed-event* effects (Luhmann et al., 2021, p. 114) and to understand specific effects of individuals’ *first* childbirth, first-time parents (treatment group; TG) were compared separately with two control groups (CGs; see Krämer and Rodgers, 2020):

1. CG1: *N* = 1769 (N_♀_ = 814) childless individuals (without any birth event until at least 308 days after their W20 interview)
2. CG2: *N* = 138 (N_♀_ = 71) parents who had an additional child (with birth events occurring before and during the event period).

#### 2.3.3 Multiple propensity score matching analyses

For both TG–CG contrasts and each sex group, we equated the study groups on *propensity scores* (PSs) using covariates measured at W17.

Since covariate selection has been described as one of the greatest challenges in PS matching (Steiner et al., 2010), we repeated all analyses with six different sets of potential confounders, following the recommendations of Rohrer (2018). Individuals with missing values on any of the matching variables used were excluded from the matching procedure.

We evaluated covariate balance as described in Supplement 3. However, the small number of CG2 candidates left little room for the matching procedure to reduce imbalance between TG and CG2 samples.

The matching process is documented in Supplement 3, including a list of covariates.

#### 2.3.4 Subsamples for final analyses

The matching design yielded 24 combinations of the two TG–CG contrasts, two sex groups, and six covariate selection sets. One subsample was excluded because the covariates did not contribute to predicting TG membership. We therefore performed final analyses separately for the remaining 23 samples (see Supplement 4).

### 2.4 Statistical analyses

For all analyses, the significance level was set at *α* = 0.05. All analyses are detailed in Supplements 5, 6.

#### 2.4.1 Manifest analyses

As a first step, we studied participants’ spiritual development using manifest means of their seven spirituality item scores.

##### 2.4.1.1 *t*-tests examining first-time parents’ intraindividual change

We conducted *t*-tests for dependent samples to compare first-time parents’ average pre- (W17) and post-birth (W20) spirituality. We also report manifest retest correlations as indicators of three-year temporal stability.

##### 2.4.1.2 *t*-tests to compare first-time parents’ and controls’ intraindividual change

To compare the TG’s mean intraindividual change with that of the CGs, we performed *t*-tests for independent samples on the study groups’ mean intraindividual change scores across the 23 matched samples.

#### 2.4.2 Latent analyses

We additionally estimated structural equation models to correct for measurement error and to separate unsystematic measurement error from systematic change when calculating retest correlations as stability measures (Eid and Kutscher, 2014). These latent analyses are described in Supplement 6.

## 3 Results

Below are the main findings of this study. Additional results, including most findings from the latent analyses, are provided in Supplement 7.

### 3.1 First-time parents

#### 3.1.1 Sample characteristics

Of the N_♀_ = 69 female and N_♂_ = 69 male first-time parents, most were Swiss (♀: 94.2%, ♂: 95.7%), lived in a partnership (♀: 98.6%, ♂: 91.3%), and belonged to a religious denomination (♀: 89.9%, ♂: 85.6%). On average, they were approximately 30 years old, with women being slightly younger on average and more homogeneous in age (M = 29.1, SD = 5.3) than men (M = 32.4, SD = 8.6).

#### 3.1.2 Manifest intraindividual change

On average, first-time parents’ mean spirituality scores were below the midpoint of the scale at baseline (♀: M_W17_ = 7.92; ♂: M_W17_ = 6.91) and did not change significantly after childbirth (i.e., comparing both measurement occasions within individuals; ♀: M_W20_ = 7.75, t(52) = −0.46, *p* = 0.645, d = −0.06, 95% CI [−0.29; 0.18]^4^; ♂: M_W20_ = 6.49, t(53) = −0.02, *p* = 0.983, d = 0.00, 95% CI [−0.24; 0.23]).

#### 3.1.3 Manifest stability

Individual spirituality mean scores’ retest correlations were r_TG♀_ = 0.87 (95% CI [0.79;0.92]) for first-time mothers and r_TG♂_ = 0.92 (95% CI [0.86;0.95]) for fathers, indicating very high stability of individual differences over the three-year study interval. This implies very low interindividual differences in intraindividual change.

#### 3.1.4 Manifest variability

Interestingly, first-time mothers’ variance of manifest spirituality scores increased slightly over time (SD_W17♀_ = 3.73; SD_W20♀_ = 4.42), while fathers’ variance did not (SD_W17♂_ = 4.38; SD_W20♂_ = 4.29).

### 3.2 First-time parents compared to controls

Before comparing the TG’s and matched CGs’ spiritual development, we evaluated the matching procedure.

#### 3.2.1 Matching success

As shown in Table S4 in Supplement 7, estimating PSs using all covariates (selection 6) yielded the highest effect sizes (0.475 ≤ R^2^_Nagelkerke_ ≤ 0.790 compared to 0.228 ≤ R^2^_Nagelkerke_ ≤ 0.276 in selection 1), but also the smallest samples owing to non-missingness requirements. Naturally, PS overlap was more satisfying when logistic regression predicted group membership less well.

Balance was satisfactory for many covariates already prior to matching (possibly due to the representative sampling procedure) and after matching, most covariates were balanced. Only (plausible) time- and age-related differences between the TG and CG2 were not equated, given the small number of CG2 candidates. Some partnership- and family-related covariates were not balanced between female TG and CG1 members when not used as matching variables themselves.

Taken together, matching was acceptable or satisfactory across all samples and yielded 23 samples comprising 62 < n_matched_ < 122 TG and CG participants in equal proportions. Sample characteristics are shown in Supplement 7.

#### 3.2.2 Difference in manifest intraindividual change

Table 1 shows TG and CG members’ average baseline manifest spirituality and three-year spiritual change scores. The latter ranged within half a scale unit across all groups (−0.33 < Δ_TG♀_ < 0.36; −0.33 < Δ_TG♂_ < 0.28; −0.52 < Δ_CG♀_ < 0.02; −0.48 < Δ_CG♂_ < 0.23) and did not differ between first-time parents and controls in either sample (*p* > 0.05). Effect sizes were small to medium (Cohen, 1988). Notably, average spirituality scores slightly declined in two female and all male CG2 samples.

**Table 1:** *t*-tests on study groups’ average intraindividual spiritual change scores.

S.^a^	Sel.^b^	TG			CG			Δ_Δ_^e^	*t*(*df*)	*p*	*d* [95% CI]^f^
		*n*	W17^c^	Δ_W20_^d^	*n*	W17^c^	Δ_W20_^d^				
F1	1	51	8.08	−0.07	57	7.98	0.02	−0.08	−0.17(106)	0.865	−0.03, [−0.41;0.34]
	2	50	8.12	−0.08	53	8.10	−0.29	0.21	0.48(101)	0.632	0.09, [−0.29;0.48]
	3	49	8.21	0.02	55	8.01	−0.48	0.50	1.16(102)	0.249	0.23, [−0.16;0.61]
	4	51	8.08	−0.07	55	7.71	−0.24	0.17	0.41(104)	0.682	0.08, [−0.30;0.46]
	5	39	8.67	0.07	43	8.32	−0.15	0.22	0.43(80)	0.671	0.09, [−0.34;0.53]
	6	37	8.82	0.13	41	7.80	−0.42	0.55	1.12(76)	0.265	0.25, [−0.19;0.70]
F2	1	51	8.08	−0.07	51	7.99	−0.15	0.08	0.18(100)	0.860	0.04, [−0.35;0.42]
	2	38	8.44	−0.33	39	7.69	−0.52	0.19	0.45(75)	0.655	0.10, [−0.35;0.55]
	3	49	8.21	0.02	51	7.59	−0.40	0.43	1.08(98)	0.281	0.22, [−0.18;0.61]
	4	51	8.08	−0.07	51	8.24	−0.28	0.21	0.45(100)	0.651	0.09, [−0.30;0.48]
	5	33	9.00	0.14	36	7.92	−0.26	0.40	0.67(67)	0.504	0.16, [−0.31;0.63]
	6	27	8.96	0.36	28	8.64	−0.46	0.82	1.45(53)	0.153	0.39, [−0.14;0.92]
M1	1	53	6.74	0.03	57	6.57	0.21	−0.18	−0.44(108)	0.660	−0.08, [−0.46;0.29]
	2	51	6.89	0.02	48	6.54	−0.34	0.36	0.82(97)	0.416	0.16, [−0.23;0.56]
	3	50	6.78	−0.01	50	6.84	−0.15	0.14	0.35(98)	0.729	0.07, [−0.32;0.46]
	4	53	6.74	0.03	56	7.34	−0.31	0.35	0.85(107)	0.396	0.16, [−0.21;0.54]
	5	44	7.11	−0.03	44	6.24	−0.27	0.24	0.66(86)	0.514	0.14, [−0.28;0.56]
	6	41	7.29	−0.02	41	7.45	0.23	−0.25	−0.61(80)	0.545	−0.13, [−0.57;0.30]
M2	1	52	6.63	0.02	49	6.10	−0.26	0.28	0.84(99)	0.402	0.17, [−0.22;0.56]
	2	44	7.30	−0.33	39	6.48	−0.45	0.12	0.36(81)	0.720	0.08, [−0.35;0.51]
	3	44	6.47	0.03	42	6.12	−0.47	0.49	1.32(84)	0.189	0.29, [−0.14;0.71]
	4	41	6.21	0.28	39	6.38	−0.48	0.76	1.97(78)	0.052	0.44, [−0.00;0.88]
	6	28	6.77	0.18	26	5.80	−0.46	0.64	1.34(52)	0.185	0.37, [−0.17;0.90]

For the sake of consistency across columns, the table only presents information on individuals with valid values for both waves’ spirituality means.

a Sample.

b Matching variable selection.

c Average manifest spirituality score at W17.

d Average intraindividual change in manifest spirituality scores from W17 to W20 (values above 0 represent increases over time).

e Difference-in-difference scores between study groups (values above 0 represent higher mean spiritual change scores in the TG than in the CG).

f Cohen’s d with a 95% confidence interval.

#### 3.2.3 Manifest and latent stability

Manifest retest correlations were 0.68 < r_CG1♀_ < 0.90 in childless women, 0.77 < r_CG1♂_ < 0.92 in childless men, 0.83 < r_CG2♀_ < 0.93 in experienced mothers, and 0.92 < r_CG2♂_ < 0.94 in experienced fathers. Likewise, latent spirituality was highly stable in first-time parents (ρ_W17W20_ ≥. 95) and in nearly all CGs, suggesting that intraindividual change between W17 and W20 was interindividually homogeneous (Eid and Kutscher, 2014). Note that manifest stability coefficients were smaller due to measurement error.

#### 3.2.4 Further latent analyses

The latent analyses fully support the findings at the manifest level (see Supplement 7).

## 4 Discussion

In this study, we aimed to obtain precise, robust, and potentially causal insights into how the birth of individuals’ first child affects their spirituality.

Following a common definition, we operationalised spirituality as desire directable to different (i.e., religious and non-religious) entities (Pargament et al., 2013).

Using longitudinal data from the SHP, representative of Switzerland’s inhabitants (Voorpostel et al., 2022), we compared first-time mothers’ and fathers’ pre- to post-birth spiritual development with that of PS-matched childless controls and experienced parents who had had another child.

To ensure that the findings were not artefacts of the choice of covariates used for PS estimation, we repeated the matching procedure using six different covariate selection sets (Rohrer, 2018) and performed our analyses on each of the 23 resulting matched samples. Matching success varied: In most matched samples, covariates were very well balanced, while in others, balance was less satisfactory. This underlines the importance of such robustness checks using different covariate selection sets. Importantly, the results were highly consistent across the different samples, all leading to the same following substantive conclusions.

### 4.1 Insights into the stability and change of spirituality following childbirth

#### 4.1.1 No average medium-term change and high stability in first-time parents

Across robustness conditions, first-time parents’ spirituality was highly stable over the three-year study period and did not change on average from pre- to post-birth, neither in absolute terms nor compared to childless and experienced parent controls.

This is consistent with evidence on well-being stability following childbirth (Bühler et al., 2024; Krämer and Rodgers, 2020) and on early adulthood as a period of increasing personality stability (Bleidorn et al., 2022).

#### 4.1.2 Slight variance increases among first-time mothers

Despite this average stability, variance slightly increased among first-time mothers. Increases in variance in TGs are commonly observed in psychotherapy research (Bergin, 1963). Here, this pattern may imply that childbirth *can* lead to lasting changes in spirituality in a positive or negative way, highlighting the need for further research on moderators and differential trajectories.

#### 4.1.3 Small declines in spirituality in some multiparous parents

Interestingly, average spirituality declined in several samples of multiparous parent controls (CG2). Although significant, these effects were small and may be age-related rather than event-related because matching did not equate multiparous and first-time parents on age. However, especially given the small sample sizes, these declines should motivate further investigations.

#### 4.1.4 Main learnings

Taken together, our manifest and latent mean-level results suggest no *average* spiritual change in first-time parents caused by childbirth. From our study’s medium-term perspective, no general trend, such as a general increase or decline in spiritual experiences and behaviors, could be observed.

However, medium-term stability does not imply that participants’ spirituality was stable throughout the entire three-year period. Given this study’s coarse temporal resolution, it cannot be ruled out that individuals experienced short-term spiritual changes around childbirth or perceived the event itself as spiritual, which is highly likely according to the literature (e.g., Callister, 2004). Nonetheless, when viewed over a three-year period, spirituality was extremely stable.

It is also important to note that mean-level stability does not imply that no participant’s spirituality changed. Increases in variance among first-time mothers and average declines in several control group samples indicate that lasting spiritual changes *did* occur for some individuals. Hence, childbirth seems to hold spiritually transformative potential for some individuals. However, the very high retest correlations suggest that such interindividual differences in intraindividual medium-term spiritual change following childbirth only occurred to a limited extent.

By design, the present study was unable to shed light on what drives differential spiritual trajectories following childbirth, that is, on whose spirituality changes and when.

Future research should scrutinise the underlying psychological mechanisms by examining mediators and moderators, taking a wide range of personal and situational variables into account. Whether individuals’ spirituality changes after childbirth might depend on their postpartum mental health, personality, risk aversion, anxiety, religious affiliation, religious service attendance, the course of birth, birth complications, pain during birth, social support, partnership situation, pregnancy intentionality, child desirability, or cultural factors, to name just a few.

### 4.2 Methodological insights

This study also provides some methodological insights regarding causal inference in quasi-experimental designs.

Firstly, we confirm that robustness checks regarding covariate selection are highly advisable (Rohrer, 2018).

Secondly, logistic regression models using many (or highly discriminating) covariates tended to predict PSs more accurately. Ironically, estimating PSs too well, that is, yielding highly precise or deterministic predictions of group membership, risked to cause separability and reduced overlap, which are threats to matching.

Finally, numerous covariates were already balanced before matching, potentially due to the representative sampling design (Voorpostel et al., 2022). Using representative data, matching only on highly relevant covariates might improve balance wherever it is most needed. For a comprehensive elaboration on causal-analytical methodology for life-event research, see Lawes et al. (2025).

### 4.3 Limitations

Despite the consistency of the findings across robustness conditions, several limitations restrict their generalisability:

First, our results can only be generalised to the population of Switzerland, a culturally heterogeneous country that remains relatively religious but is increasingly secular (cf. Schweizer Radio und Fernsehen, 2024). Individuals do not develop their spiritual reactions to life events in a vacuum; they are shaped by their cultural surroundings. Therefore, results might be very different in other countries.

Second, as noted earlier, the long time span between measurements only allowed for medium-term statements. Assessing spirituality more frequently and in closer proximity to childbirth events would enlighten the complex meaning-making processes during the multifaceted transition to parenthood and would allow for detecting short-term fluctuations, as well as for distinguishing childbirth from frequently co-occurring life events (see Krämer et al., 2025). In particular, future research should aim to disentangle direct childbirth effects from indirect effects mediated by changes in religiosity. Such changes may occur following initiation rites such as baptism, which parents may celebrate with their newborn. In addition, research suggests that some young adults begin or intensify their attendance at religious services after becoming parents (e.g., Uecker et al., 2016).

Third, using a national panel resulted in relatively small and homogeneous samples and a limited set of variables, which impeded explorations of cultural differences or situational moderators of spiritual change and constrained the application of more sophisticated matching procedures (see Krämer and Rodgers, 2020). Especially between the equally small groups of nulliparous and multiparous parents, matching was almost unable to improve covariate balance. Given the very small effect sizes observed in our study, we conducted several robustness checks. We ran our analyses separately for two sex groups, two types of control groups, and different sets of covariates used for PS estimation, resulting in 23 robustness conditions in total, which may be seen as a form of replication. As results were similar across robustness conditions and effect sizes’ confidence intervals covered small effects, our results seem robust despite the small sample sizes. While our study’s results can be generalised to the Swiss population due to representative sampling, its estimated effect sizes may serve as useful benchmarks for sample size planning in future studies targeting specific samples.

Finally, due to data availability, we applied a binary gender model and focused on biological parenthood. However, it cannot be ruled out that members of the “childless” control groups adopted children during the event period. Life-event research and survey methodology must urgently evolve to better reflect the numerous pathways to becoming a parent today.

## 5 Conclusion

Taken together, in this first study using representative panel data and causal inference methods, spirituality appeared remarkably stable in the medium term and on average, across robustness conditions involving women and men, first-time and experienced parents, and childless controls.

The present study may serve as a foundation for future studies that delve into the psychological mechanisms underlying spiritual stability and change following childbirth. It also provides a methodological framework for this type of research, and its effect sizes may be useful for power analyses.

Contributing to a growing body of literature on spirituality (see Davis et al., 2023), future research should not only focus on differential trajectories and moderating situation characteristics in addition to average effects and explore the extent to which spirituality’s development over the lifespan is predetermined versus reactive to other, perhaps more igniting, life events such as wars and pandemics. Future studies should also continue to examine how spirituality itself transforms people’s lives.

## Data Availability

Publicly available datasets were analyzed in this study. This data can be requested free of charge from the FORS Centre at: https://forscenter.ch/projects/swiss-household-panel/data/.

## Footnotes

1 Swiss Household Panel data access can be requested free of charge from the FORS Centre at: https://forscenter.ch/projects/swiss-household-panel/data/.

2 This information was only used in one direction: The presence of a linked child’s birth date assured that a birth event had occurred – its absence could also be due to missingness or because parents might have not agreed to add their children to the panel.

3 This study was not preregistered because results were partially known *a priori* from the master’s thesis this study is based on.

4 Because normality of the difference variable could not be assumed, a *Sign Test* was additionally conducted. It led to the same conclusion.
