# Supplementary material for: Stability and change of spirituality following childbirth: longitudinal evidence from Swiss Household Panel data using multiple propensity score matching analyses
Source: Front Psychol. 2026 Aug 4;17:1813504. doi: 10.3389/fpsyg.2026.1813504 (PMC13481443; doi:10.3389/fpsyg.2026.1813504)
Supplement: Supplementary file 1 [file Supplementary_file_1.pdf]

Stability and Change of Spirituality Following Childbirth: Longitudinal Evidence from Swiss Household Panel Data Using Multiple Propensity Score Matching Analyses

Supplemental Material

Friedemann Trutzenberg* & Michael Eid
Department of Education and Psychology, Freie Universität Berlin, Berlin, Germany

July 14, 2026

*friedemann.trutzenberg@fu-berlin.de

Contents

Supplement 1: Panel search 4

Supplement 2: Spirituality items 5

Supplement 3: Matching 6
- Preconditions for matching . . . 6
- Matching variable selections . . . 6
  - Matching variable selection 1: Basic . . . 6
  - Matching variable selection 2: Demographic . . . 7
  - Matching variable selection 3: Partner/Family . . . 7
  - Matching variable selection 4: Well-being . . . 8
  - Matching variable selection 5: Personality . . . 8
- Propensity score estimation using logistic regression . . . 14
  - Model selection procedure . . . 14
  - Regression diagnostics . . . 14
  - Models' predictive power . . . 14
- Propensity score matching . . . 15
  - Matching procedure . . . 15
  - Evaluation of matching success . . . 15

Supplement 4: Overview of subsamples for final analyses 16

Supplement 5: Statistical analyses I: Manifest analyses 17
- *t*-tests to study intraindividual change in first-time parents . . . 17
- *t*-tests to compare TG and CG members' intraindividual change . . . 17

Supplement 6: Statistical analyses II: Latent analyses 18
- Item parceling . . . 18
  - Theoretical considerations . . . 18
  - Polychoric correlations . . . 18
  - Parceling rationale . . . 21
  - Parcel overview . . . 21
- Model estimation . . . 22
  - Robust Maximum Likelihood estimator . . . 22
  - Full information maximum likelihood approach . . . 22
- Model fit criteria . . . 22
- Latent state models . . . 23
  - Factor structure . . . 23
  - Strict measurement invariance . . . 23
  - Scale of latent means . . . 23
- Latent change models . . . 25
  - Factor structure . . . 25
  - Strict measurement invariance . . . 25
  - Scale of latent means . . . 25
- Comparison of study groups' latent change . . . 27
  - Models with additional restrictions . . . 27
  - Model comparison . . . 27

Supplement 7: Results 28
- Propensity score matching . . . 28
  - Overall matching success . . . 28
- Matched sample characteristics . . . 29
- Manifest matching-based analyses . . . 33

t-tests for independent samples . . . 33
Manifest retest correlations . . . 33
Latent matching-based analyses . . . 35
Latent state models . . . 35
Latent change models . . . 35
Central findings from latent state and latent change models . . . 35

References **44**

Supplement 1: Panel search

To find the data base most suitable for the aim of the present study, the eligibility of over 90 panel studies from different countries was evaluated. Two of these studies assessed more than two spirituality items in at least region-wide representative adult samples at more than one measurement occasion allowing to compare pre- and post-pregnancy spirituality. With its data from 46 countries, the religion module of the *International Social Survey Programme* (ISSP; ISSP Research Group, 1993, 2000, 2018, 2020) is a promising foundation for future research. The *Swiss Household Panel* (*SHP*; Voorpostel et al., 2022) also fulfilled all eligibility criteria. As we aimed to establish our methodology based on numerous careful robustness checks in one sample first and because a cross-cultural comparison study would thus have entailed complex supplementary analytical challenges, the SHP was chosen as data source for the current study.

Supplement 2: Spirituality items

Table S1 shows the seven SHP items used to measure spirituality in the current study.

Table S1: Spirituality items

No.	Item	Question	Cat.[a]
1	Feeling of spirituality	Putting aside whether or not you would describe yourself as a religious person, how spiritual would you say you are?[b]	5[c]
2	Belief in God or something divine	To what extent do you believe in God or something divine?[d]	5[c]
3	Feeling to be at one (frequency)	How often do you experience situations where you have the feeling that you are at one?[ed]	5[f]
4	Feeling that God intervenes in life (frequency)	How often do you experience situations where you have the feeling that God or something divine intervenes in your life?[d]	5[f]
5	Think about religious questions (frequency)	How often do you think about religious questions?[dg]	5[f]
6	Prayers apart from church or religious community (frequency)	How frequently do you pray apart from at church or within a religious community?[h]	6[i]
7	Meditation (frequency)	How often do you meditate?[d]	6[i]

Note. [a]Number of response categories; [b]Stolz & Monnot, as cited in SHP Group, 2022, individual questionnaires of W17 and W20; [c]Response categories of items 1 and 2 after rescaling: 0: not at all - 5: a little / not very much - 10: moderately / medium - 15: quite / fairly - 20: very (much); [d]Huber, 2008; Huber et al., 2011; see SHP Group, 2022, individual questionnaires of W17 and W20; [e]Additional instruction for item 3: 'For instance moments when you feel completely in harmony with nature'; [f]Response categories of items 3, 4 and 5 after rescaling: 0: never - 5: rarely - 10: occasionally - 15: often - 20: very often; [g]Additional instruction for item 5: '"Religious questions" in a broad sense; spiritual questions are also included.'; [h]Huber, 2008; Huber et al., 2011; see SHP Group, 2022, individual questionnaire of W17; [i]Response categories of items 6 and 7 after rescaling: 0: never - 4: a few times a year - 8: about once a month - 12: at least once a week - 16: daily or almost daily - 20: several times a day.

From the SHP individual questionnaires of W17 and W20 published in: SHP Group (2022). Living in Switzerland Waves 1-22 + Covid 19 data [Data set]. *FORS - Swiss Centre of Expertise in the Social Sciences.* https://doi.org/10.48573/1nav-wy98
Reprinted with permission. © FORS - Swiss Centre of Expertise in the Social Sciences.

According to the SHP documentation distributed together with the data (SHP Group, 2022, individual questionnaires of W14, W17 and W20), the first item was included on the recommendation of Christophe Monnot and Jörg Stolz from the University of Lausanne, Switzerland, and the University of Strasbourg, France.

The items 2, 3, 4, 5, 6 and 7 take a bearing on items originating from the Centrality of Religiosity Scale (Huber & Huber, 2012).

For their use in the SHP, some items were slightly adapted. The items aim at a concept broader than religiosity, in line with the definition of spirituality outlined in the article (Huber & Huber, 2012; SHP Group, 2022, individual questionnaires of W14, W17 and W20).

Supplement 3: Matching

In the following, we will present and justify the selections of variables used for matching, describe our procedures to calculate individuals' propensity scores based on logistic regression and explain how we matched our study groups on these propensity scores.
We conducted all analyses using *R* (version 4.2.2; R Core Team, 2022).

Preconditions for matching

We considered the *stable unit treatment value assumption* (SUTVA; West et al., 2014) to be met given the SHP's Switzerland-wide recruitment strategy (Voorpostel et al., 2022) and the separation by gender, making it unlikely that sample members had contact to one another.

As the matching variables were measured *before* the treatment and the outcome, we could rule out they were *colliders* or *mediators* (Rohrer, 2018).

Matching variable selections

In the following, we present the variables that we used during the matching process. All matching variables were assessed at W17 (see Figure 1). Table S2 gives an overview which variables we used, how we transformed them and in which matching variable selection they were included.

For propensity score matching, only individuals with valid values on all covariates matched on were considered (D'Agostino et al., 2001, as cited in Stuart & Rubin, 2007), reducing our matched samples' sizes. As most missing values were unavailable by panel design (i.e., the participants had not been presented with the concerning items during their interviews), we assumed that missingness was not due to participants' unobserved values on the variables in question.

Matching variable selection 1: Basic

As shown in Table S2, the first matching variable selection contained the variables *Age (in years)* and *Spirituality at W17 (manifest mean).* These two highly relevant potential confounders were also included in the second to fifth matching variable selections which additionally contained one additional semantic set of matching variables each.

This was done to balance TGs and CGs on their age and baseline spirituality in all matched samples.

Age was measured in years. *Spirituality at W17 (manifest mean)* was computed as mean value of the seven spirituality variables measured at baseline. Due to its semantic relevance, we included this measure despite some missing values.

We centered both variables.

Matching variable selection 2: Demographic

Because parents in Switzerland have been found to show specific distributions both on demographic and on socio-economic variables (Bundesamt für Statistik [BFS], 2015, p. 2, 2017, p. 32, 2019, 2020a, 2020b) and because demographic and educational variables have been proven to be correlated with perceived spiritual changes after childbirth (Berman et al., 2020, p. 4; Rozen et al., 2018, p. 2178), we have selected the demographic variables shown in Table S2 into the second matching variable selection. Further, semantically similar demographic variables were available in the SHP (see SHP Group, 2022, individual questionnaires of W17). However, we carefully created the aforementioned selection to avoid multicollinearity (see Eid et al., 2015, p. 823).

We regrouped the categorical variables *Denomination* and *Occupational status* to make them coarser and to avoid yielding too small groups or zero cells. *Highest education* was coded using the *International Standard Classification of Education* (ISCED; United Nations Educational, Scientific and Cultural Organization [UNESCO], 2003). We reordered the variable to make it fully ordinal. Participants' first nationality was dichotomized into Swiss vs. non-Swiss. *Yearly net wage* was measured in CHF. We decided to include it despite its proportion of missing values because of its role as central demographic variable. We regrouped and reordered the variable *Rurality* into large centres, other uniquely urban municipalities, mixed municipalities and uniquely rural municipalities in line with a spatial typology of urbanity and rurality published by the Swiss Federal Office for Spatial Development (2005, p. 10).

We centered all continuous variables.

Matching variable selection 3: Partner/Family

Similarly to demographic factors, variables on household and family life, too, seem to be distributed in a specific way in Swiss parents (BFS, 2016, 2017, p. 32). To avoid that TG and CG would largely differ with regard to this life domain, we have selected the partnership and family variables shown in Table S2 into the third matching variable selection.

We regrouped the categorical variable *Civil status* to make it coarser and to avoid yielding too small groups or zero cells. We counted registered partnerships as marriages and ends of registered partnerships as divorces. Participants' *Partnership status* at W17 needed to be inferred using different original SHP variables because depending on information from the household interviews, direct information on partnership was only collected from some individuals: In this study, we considered SHP participants to live in a relationship when they indicated they did or had a valid value larger than 0 on the variable *duration of partnership*. The *Duration of partnership* was measured in years. We transformed this variable so that participants not living in a relationship received a 0. We inferred individuals' *Household constellation* from the original SHP variables *Satisfaction with living alone* and *Satisfaction with living together*, depending on which of the two variables individuals had a missing value. Participants' *Happiness with household constellation* was computed combining the same two variables on which participants only had one valid value. Values of the variable *Minimum number of desired children* equaled the values of the original SHP item *Total number of children wanted* whenever the latter was not missing and 1+ the *Number of children born* at W17 whenever the *Total number of children wanted* was missing and the binary original SHP item *Child wanted in the next 24 months* had the value "yes". This transformation was able to reduce the number of missing values but did not eliminate them completely. Nevertheless, we decided to include the *Minimum number of desired children* as a matching variable due to its potential predictive relevance.

We centered all continuous variables.

Matching variable selection 4: Well-being

Apart from variables on demography, socio-economic status, household and family life, several psychological variables are discussed as predictors of childbirth: In a study using longitudinal data from three panel studies cited above, Luhmann and colleagues (2013) found that higher life satisfaction prospectively increased the likelihood of childbirth (p. 42). Little is known about such factors' influence on spiritual change (see above). Therefore, to avoid missing out unknown confounder variables, we have selected the health, well-being and self-perception variables shown in Table S2 into the fourth matching variable selection.

We included the variable *Health status* to make sure that TG and CG members would not differ in their overall health, which could be related to their fertility. We computed participants' *Life satisfaction* (cognitive well-being) using the mean of five original variables on life satisfaction originating from the Satisfaction with Life Scale (Diener et al., 1985, as cited in SHP Group, 2022, e.g. individual questionnaire of the wave 20, pp. 29-33). Calculating a sum or mean score of the scale has been proposed by Pavot and Diener (2008, p. 141), however, even studies investigating the scale's factor structure (cf. Lewis et al., 1995) do not report whether the requirements of essential τ equivalence, uncorrelated error variables and equal error variances, are met (Steyer & Eid, 2001, pp. 129-130). We determined *Positive Affect* using the mean of two original SHP variables based on general and emotion-specific conceptualisations of affective well-being (Scherer et al., 2004; Watson et al., 1988, both cited as in Voorpostel et al., 2022, pp. 63). *Negative affect* was computed as the mean of the four negatively coded variables from the same sources (ibid.). We used a scale proposed by Voorpostel and colleagues (2022, p. 65) as general measure on self-related attitudes similar to the concepts of self-efficacy (see Bandura, 1977) and locus of control (Rotter, 1966). The scale consists of twelve SHP items from different sources (Lachman & Weaver, 1998; Levy et al., 1997, p. 510; Pearlin & Schooler, 1978; Rosenberg, 1965, all as cited in Voorpostel et al., 2022, p. 65) and the SHP user guide (Voorpostel et al., 2022, p. 65) reports an internal consistency of .74 on W14 SHP data. We computed the items' mean value and called the resulting matching variable *Self-perception / Self-efficacy / Locus of Control*. However, as was the case with the Satisfaction with Life Scale (Lewis et al., 1995), no essential τ equivalence has been reported in the literature so that it remains unclear whether all preconditions for the use of such a mean score are met (cf. Steyer & Eid, 2001).

We centered all continuous variables.

Matching variable selection 5: Personality

Finally, to be able to balance TG and CG personality variables, we have selected the Big Five personality traits into the fifth matching variable selection as shown in Table S2. Personality has been found relatively stable over time (Cobb-Clark & Schurer, 2012; Rantanen et al., 2007, pp. 515-516) and is associated with spirituality and religiosity (Schnell, 2012, pp. 44-45). In turn, Beck and Jackson (2022) report higher likelihoods for childbirth due to higher extraversion and conscientiousness (p. 536) on the basis of matched data from several panel studies in a "Mega-Analytic" framework.

The Big Five have been measured at W17 using the Big Five Inventory-Short Version (BFI-15; Gerlitz & Schupp, 2005, as cited in Voorpostel et al., 2022, p. 64). For each of the five subdomains, we used the three respective items' mean value as matching variable.

We centered all five variables.

Table S2: Overview of matching variable selections

**Selection**/Variable	**Ref. cat. / Scale**[a]	**Original SHP item(s)**	**Transformations**
**1. Basic**			
Age (in years)		• Age in year of interview	
Baseline spirituality		• Feeling of spirituality<br>• Belief in God or something divine<br>• Feeling to be at one: frequency<br>• Feeling that God intervenes in life: frequency<br>• Think about religious questions: frequency<br>• Prayers apart from church or religious community: frequency<br>• Meditation: frequency	Rescaled, then mean value
**2. Demographic**			
Age (in years)		*See above*	*See above*
Baseline spirituality		*See above*	*See above*
Denomination	"none"	• Confession or religion	Regrouped
Education	0-60 (higher values: higher level of education)[b]	• International Standard Classification of Education ISCED 1997	Reordered
Interview language	"German"	• Interview language	
First nationality	"Swiss"	• First nationality by region	Dichotomised
Yearly wage		• Yearly total personal income, net	
Occupational status	"full"	• Actual occupation, from grid	Regrouped
Region	"Middleland"	• Region of residence	
Rurality	1-4 (higher values: greater rurality)[c]	• Community typology 1	Regrouped, reordered

**Selection**/Variable	**Ref. cat. / Scale**[a]	**Original SHP item(s)**	**Transformations**
**3. Partner/Family**			
Age (in years)		*See above*	*See above*
Baseline spirituality		*See above*	*See above*
Civil status	"single"	• Civil status in year of interview	Regrouped
Partnership status	"no"	• Partner: Yes, no<br>• Partner or spouse: Since when: Corrected year	Status inferred
Partnership duration (in years)		• Partner or spouse: Since when: Corrected year	Singles' duration added
Household constellation	"living alone"	• Satisfaction with living alone<br>• Satisfaction with living together	Status inferred
Happiness with household constellation	0-10 (higher values: higher satisfaction)	• Satisfaction with living alone<br>• Satisfaction with living together	Summarised
Number of desired children		• Total number of children wanted<br>• Child wanted in the next 24 months<br>• Number of children born	Number inferred
**4. Well-being**			
Age (in years)		*See above*	*See above*
Baseline spirituality		*See above*	*See above*
Health status	1-5 (higher values: poorer health)	• Health status	
Life satisfaction	0-10 (higher values: higher satisfaction)	• Satisfaction with live in general<br>• LS: Life close to ideal<br>• LS: Excellent life conditions<br>• LS: Having gotten important things<br>• LS: Not changing anything	Mean value
Positive affect	0-10 (higher values: higher frequency of positive emotions)	• Frequency of energy and optimism<br>• Emotions: Joy: Frequency	Mean value

**Selection**/Variable	**Ref. cat. / Scale**[a]	**Original SHP item(s)**	**Transformations**
Negative affect	0-10 (higher values: higher frequency of negative emotions)	• Depression, blues, anxiety: Frequency<br>• Emotions: Anger: Frequency<br>• Emotions: Sadness: Frequency<br>• Emotions: Worry: Frequency	Mean value
Self-perception / Self-efficacy / Locus of Control	0-10 (higher values: higher sense of control)	• Self-Perception: incapacity to make plans because of unpredictability<br>• Self-Perception: little influence on life events<br>• Self-Perception: capacity to overcome unexpected problems<br>• Self-Perception: capacity to choose between two possibilities<br>• Self-Perception: feeling of uselessness<br>• Self-Perception: feeling of selfsatisfaction<br>• Sense of control: Doing everything set in my mind<br>• Sense of control: Find a way to succeed<br>• Sense of control: What I want is in my hands<br>• Sense of control: What will happen depends on me<br>• Sense of control: Others determine what I can do<br>• Sense of control: Feeling of being pushed in my life	Inverted items recoded, then mean value

**Selection**/Variable	**Ref. cat. / Scale**[a]	**Original SHP item(s)**	**Transformations**
**5. Personality**			
Age (in years)		*See above*	*See above*
Baseline spirituality		*See above*	*See above*
Openness	0-10 (higher values: higher openness)	• Big five inventory 15: original<br>• Big five inventory 15: artistic experiences<br>• Big five inventory 15: imagination	Inverted items recoded, then mean value
Conscientiousness	0-10 (higher values: higher conscientiousness)	• Big five inventory 15: does a thorough job<br>• Big five inventory 15: lazy<br>• Big five inventory 15: efficient	Inverted items recoded, then mean value
Extraversion	0-10 (higher values: higher extraversion)	• Big five inventory 15: talkative<br>• Big five inventory 15: sociable<br>• Big five inventory 15: reserved	Inverted items recoded, then mean value
Agreeableness	0-10 (higher values: higher agreeableness)	• Big five inventory 15: rude<br>• Big five inventory 15: forgive<br>• Big five inventory 15: kind	Inverted items recoded, then mean value
Neuroticism	0-10 (higher values: higher neuroticism)	• Big five inventory 15: worry<br>• Big five inventory 15: nervous<br>• Big five inventory 15: is relaxed	Inverted items recoded, then mean value

**Selection**/Variable	**Ref. cat. / Scale**[a]	**Original SHP item(s)**	**Transformations**
**6. Best of**			
Age (in years)		*See above*	*See above*
Baseline spirituality		*See above*	*See above*
all other matching variables			

Note. The matching variables belonging to all six matching variable selections are shown. The table also shows the SHP items the matching variables originate from as well as all transformation steps. [a]Ref.cat. / Scale: This column indicates the reference category for categorical items and provides information on the scale for continuous items. [b]Coding scheme for the ordinal variable *Highest degree of education*: 0: Not completed primary (compulsory) education, 10: Primary or first stage of basic education, 20: Lower secondary or Second stage of basic education, 33: Upper secondary education (entrance into the labor market), 32: Upper secondary education (preparation for further prof. education), 31: Upper secondary education (preparation for tertiary education), 41: Post-secondary education non tertiary (preparation for an institution for higher education), 52: First stage of tertiary education (professional education), 51: First stage of tertiary education (general education), 60: Second stage of tertiary education. [c]Coding scheme for the ordinal variable *Rurality*: 1: Large centres, 2: Other uniquely urban municipalities, 3: Mixed municipalities, 4: Uniquely rural municipalities (SHP Group, 2022, household and individual questionnaires of W17).

Propensity score estimation using logistic regression

For the sake of correct specification of logistic regression models estimating PS and to avoid deterministic predictions (see West et al., 2014), we tested interaction, quadratic and cubic effects and conducted regression diagnostics.

Model selection procedure

Matching variable selections 1 to 5. Within the matching variable selections 1 to 5, an additive multiple logistic regression model was estimated on the basis of predictors' single-order polynomials.

If this model fit the data better than a null model, a model including two-way interactions of all pairs of metric predictors and effects of all metric predictors' second-order polynomials was estimated regardless of whether concerning predictors' single-order polynomials' main effects had been significant in the first model (interaction effects involving categorical predictors were omitted to avoid separability due to zero cells; see Eid et al., 2015, p. 827-828). If at least one of the interaction effects or effects of second-order polynomials was significant, a reduced model only containing significant interaction effects and significant effects of second-order polynomials was estimated (except for when they were all significant). To increase predictive precision with regard to the logistic regression models' causal analytic purpose, this reduced model also included all predictors' single-order (main) effects regardless of whether or not they had been significant in the additive single-order model.

If this (if necessary reduced) model contained effects of second-order polynomials and fit the data better than the additive single-order model, a model with third-order polynomials of all predictors with significant effects of second-order polynomials was estimated. If at least one effect of a third-order polynomial was significant, a reduced model only containing significant effects of third-order polynomials was estimated (except for when they were all significant).

If this reduced model fit the data better than the (moderated) second-order model (reduced if necessary), it was chosen for the propensity score matching process, if not, the (moderated) second-order model (reduced if necessary) was chosen. If the moderated and/or second-order model (reduced if necessary) contained no effects of second-order polynomials, it was chosen for the propensity score matching process if it fit the data better than the additive single-order model. If the moderated and/or second-order model (reduced if necessary) did not fit the data better than the additive single-order model, the additive single-order model was chosen for the propensity score matching process. If the additive single-order model did not fit the data better than a null model, the model selection process was halted and the respective matching variable selection was dropped for the given sample.

Matching variable selection 6. The matching variable selection 6 contained all predictors' single-order (main) effects and all additional effects that had been significant in at least one other selections' final model.

Regression diagnostics

Multicollinearity. For the additive single-order model, *variance inflation factors* were calculated to quantify the danger of multicollinearity. If problematic multicollinearity was detected, potentially redundant predictors were dropped and the model selection process outlined above was continued without these predictors.

Outliers and influential data points. For each selection's final model, *leverage* and *Cook's distance* values were computed to detect outliers and influential data points. If evidence for outliers or single highly influential data points was found, model parameters and standard errors of a model without these individuals were compared to the original model's estimates. Individuals were only dropped if there was overwhelming diagnostic evidence that several model parameters did only significantly differ from 0 due to their presence in the sample.

Models' predictive power

Nagelkerke's effect size was computed for each matching variable selection's final model.

Propensity score matching

Matching procedure

We performed the matching procedure using the MatchIt package (Ho et al., 2011).

The fitted values, i.e., TG membership probabilities, estimated by the matching variable selections' final logistic regression models were used as propensity scores for the matching procedures.
For each matching variable selection, nearest-neighbour 1:1 propensity score matching without replacement was performed using the selection's specific sample of participants without missing values on the selections' matching variables.

Aiming at estimating the *Average Treatment Effect of the Treated* (ATT) whenever possible, i.e., including all available TG candidates into the matched sample whenever possible (see Holland, 1986), no caliper was set and only CG candidates were discarded when their propensity score was outside the region of common support.

To ensure the distance between matching partners' propensity scores was as small as possible on average (without caliper) and no TG candidate with a very high or low propensity score was matched to a CG candidate with a very different propensity score, the matching process was conducted in random order.

Evaluation of matching success

After matching, propensity score overlap as well as propensity score and covariate balance were evaluated closely. Covariate balance was judged on the basis of standardized mean differences, variance ratios and QQ plots for continuous covariates and relative frequency differences for categorical covariates (that were treated as dummy variables). Standardised mean differences were calculated as described by Greifer (2023).
We evaluated covariate balance as proposed by Stuart and Rubin (2007).

Supplement 4: Overview of subsamples for final analyses

Table S3 shows all 23 subsamples final analyses were conducted on.

Table S3: 23 Subsamples for All Final Analyses

**Covariates**	**TG vs. CG1**		**TG vs. CG2**	
	♀: F1[a]	♂: M1[b]	♀: F2[a]	♂: M2[b]
1. Basic	F1M1	M1M1	F2M1	M2M1
2. Demographic	F1M2	M1M2	F2M2	M2M2
3. Partner/Family	F1M3	M1M3	F2M3	M2M3
4. Well-being	F1M4	M1M4	F2M4	M2M4
5. Personality	F1M5	M1M5	F2M5	[c]
6. All	F1M6	M1M6	F2M6	M2M6

Note. Age and baseline spirituality were considered indispensable matching variables and always included. A list of covariates can be found in Supplement 4.

[a]F: female. [b]M: male. [c]Note that sample M2M5 was dropped for further analyses because the covariates did not predict TG membership better than a null model in this sample.

Supplement 5: Statistical analyses I: Manifest analyses

We conducted all analyses using *R* (version 4.2.2; R Core Team, 2022).

t-tests to study intraindividual change in first-time parents

t-tests for dependent samples were conducted on the manifest level to compare TG candidates' individual manifest means of all seven items measuring spirituality between W17 (2015) and W20 (2018).
As pre- and post-birth spirituality were compared intraindividually, we considered the pre-and post-birth samples dependent.

Given the SHP's random Swiss-wide sampling strategy, we considered value pairs (i.e., individuals) independent from each other.

Before conducting the *t*-test for dependent variables, we checked whether the difference variable was normally distributed. Whenever the sample size was larger than $n = 30$ and the spiritual change scores were approximately symmetrically distributed, the *t*-test was considered robust against violations of the normal distribution assumption (Eid et al., 2015, pp. 335-336). This was also assumed whenever the sample size was larger than $n = 50$ and the spiritual change scores were only moderately skewed. When normality could not be assumed with certainty, we additionally conducted a *Sign Test* to be sure (see Eid et al., 2015, pp. 377-378).

We set the significance level to $\alpha = .05$ to test whether TG candidates' mean intraindividual changes differed significantly from 0.

We calculated Cohen's d_2'' and its 95% confidence interval to quantify effect sizes.

t-tests to compare TG and CG members' intraindividual change

In these *t*-tests, we compared the study groups' mean individual change in the manifest means of all seven spirituality items between W17 and W20.

We considered all TGs and all respective CGs independent samples for these *t*-tests because despite their similar propensity scores (and their desired similarities on numerous covariates), we assumed that matching partners did not know about one another. (Note that the *p*-value in the *t*-test for independent samples on mean change scores equals the *p*-value of the interaction effect in a two-way repeated-measures ANOVA, also referred to as split-plot analysis, when study group membership is considered a between factor because the study groups are considered independent samples.)

Whenever the sample size was $n > 30$ per group and the spiritual change scores were approximately symmetrically distributed, we considered the *t*-test robust against violations or the normal distribution assumption (Eid et al., 2015, pp. 335-336). This was also true whenever the sample size was much larger than $n = 30$ per group and the spiritual change scores were slightly skewed. The sample size was never $n < 30$ per group. We then conducted *t*-tests whenever the spiritual change scores were either normally distributed (or one of the aforementioned robustness criteria was met) and when the *Levene* test did not suggest to reject the null hypothesis of variance homogeneity between the study groups.

As the TG was the reference category of the factorised group variable (`GROUPf`) used for the *t*-tests, positive *t*-values resulted whenever the mean spiritual change score was higher in the TG than in the CG.

To quantify effect sizes, we calculated Cohen's d_2' and their 95% confidence intervals.

Supplement 6: Statistical analyses II: Latent analyses

To control for measurement error, we additionally estimated structural equation models using *lavaan* (Rosseel, 2012) in *R* (version 4.2.2; R Core Team, 2022).

As we were not aware of any published latent model measuring spirituality using all seven SHP items in the literature, the development of a new latent modelling strategy was needed and is presented below.

Item parceling

Given the comparably low sample size, latent analyses were performed on the level of parcels rather than single items. The seven spirituality items were split into three parcels following theoretical considerations. To make sure this did not remove an unnecessarily large share of systematic variance and to underpin the theoretical correlations with empirical evidence, we also considered the items' polychoric correlations for parcel creation.

Theoretical considerations

Following the conceptualisation of spirituality as outlined in the beginning of our paper, our aim was to make sure the measurement of spirituality encompassed both the religious and non-religious feelings, attitudes and behaviours. On the other hand, it had to be avoided that the measurement semantically represented religiosity and "everything that did not fit to it".

Polychoric correlations

Figures S1 and S2 show polychoric correlation coefficients between the seven SHP items on spirituality for W17 and W20 in every subsample. Polychoric correlation was used to account for the variables' ordinal response categories (Eid et al., 2015, pp. 547-548), especially the uneven distances between the response categories of the items 6 and 7 (see Table S1).

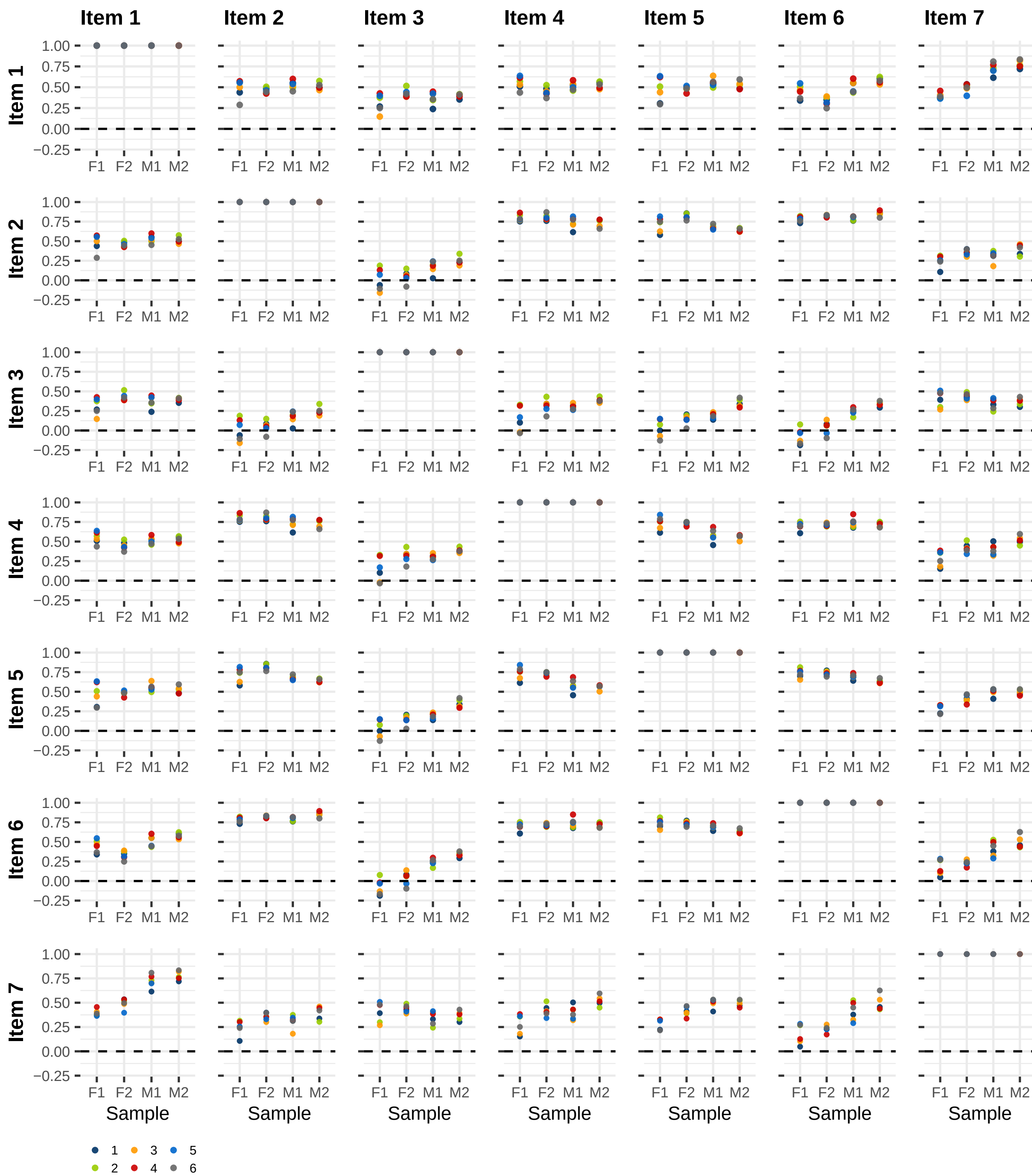

Figure S1: Polychoric correlations of the SHP items measuring spirituality at W17

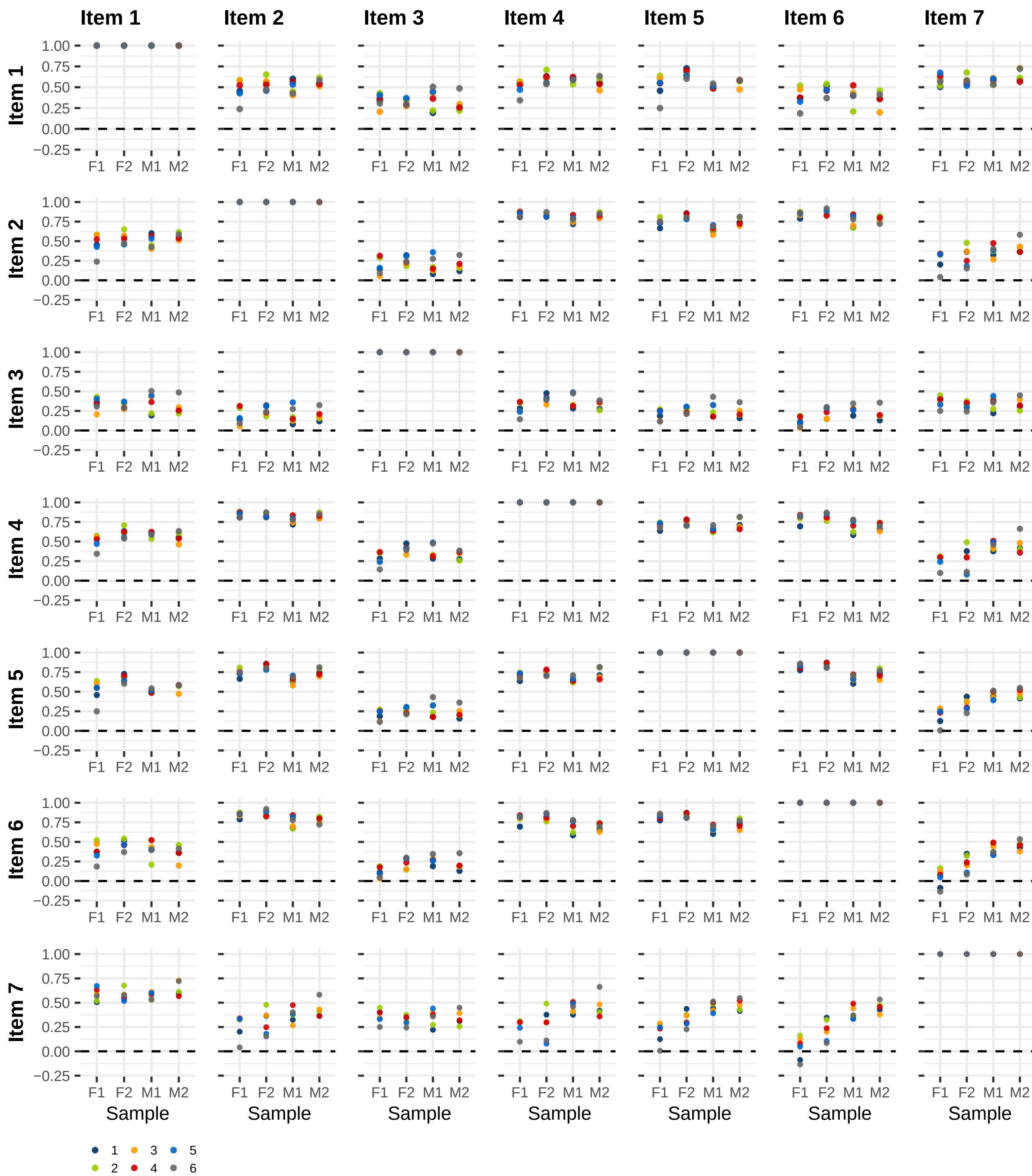

Figure S2: Polychoric correlations of the SHP items measuring spirituality at W20

Summary. Although the pattern is not entirely semantically clear, it could be assumed that the observed heterogeneous correlative pattern reflects that spirituality can be expressed both religiously and non-religiously in line with common definitions (Davis et al., 2023, p. 280; Harris et al., 2018, pp. 3, 13; Hill et al., 2000). This is supported by the fact that item 1, the general feeling of spirituality, was moderately correlated with all items in most samples.

Parceling rationale

To reflect the behaviour-centered character articulated in spirituality's most common definition as "search for the sacred" (Pargament, 1999, p. 12), all three parcels are computed using one of the behavioural items 5 (*Think about religious questions*), 6 (*Prayer; frequency*) and 7 (*Meditation; frequency*) that can all be seen as search movements.

As the definition of spirituality explicitly allows for *the sacred* to be a religious/divine or a non-religious/non-divine being and to account for the concerning items' strong associations revealed in the correlative analyses shown above, one general parcel, one parcel reflecting experiences with and behaviours towards a non-divine *sacred* and one parcel reflecting beliefs in, experiences with and behaviours towards a divine *sacred* being were constructed. Owing to the defined equivalence of divine and non-divine *sacred* beings, this adopted approach is compensatory, allowing individuals to score high on latent spirituality no matter whether their spirituality is directed to a divine or a non-divine *sacred*.

Parcel overview

Parcel 1 ("general")

- Item 1 (*Feeling of spirituality*)
- Item 5 (*Think about religious questions*; spiritual questions also included)

Parcel 2 ("non-divine")

- Item 3 (*Feeling to be at one*)
- Item 7 (*Meditation; frequency*)

Parcel 3 ("divine")

- Item 2 (*Belief in God or something divine*)
- Item 4 (*Feeling that God intervenes in life*; or something divine)
- Item 6 (*Prayer; frequency*)

Model estimation

Robust Maximum Likelihood estimator

Multivariate normal distribution tests revealed that the three parcels were not normally distributed over time in any of the matched samples. As the items underlying the parcels were not continuous but ordinal, multivariate normal distribution had not been expected, however, the tests were conducted to check for approximate normal distributions. As these were not found and as the parcels were not ordinal because they had been computed as the mean of two items each, the *Robust Maximum Likelihood* estimator (Eid et al., 2015, p. 902) was used for the analyses in this study.

Full information maximum likelihood approach

We applied the *full information maximum likelihood* approach (*fiml*) to handle missing values without losing more empirical information (Eid et al., 2015, pp. 900-901).

Model fit criteria

Whenever the null hypothesis of exact model fit did not have to be rejected (χ^2 value not significant), we assumed that the given model fit the data. However, given the models' relatively high number of parameters compared to our small sample sizes, we still considered the model fit as satisfactory when the null hypothesis of exact model fit had to be rejected, but two of the following criteria of approximative fit were met:

- $\chi^2 < 2 \cdot df$ (Schermelleh-Engel et al., 2003, p. 33)
- CFI > .97 (ibid., p. 42)
- lower limit of the RMSEA confidence interval < 0.05 (ibid., p. 37)
- SRMR < .08 (Hu & Bentler, 1999, p. 27).

If only the $\chi^2 < 2 \cdot df$ criterion was met and $CFI > .95$, we considered the model fit as improvable, but acceptable (Schermelleh-Engel et al., 2003, p. 42). Note that CFI, RMSEA and SRMR have been found sensitive to small sample sizes, the RMSEA also to small numbers of *df*, rejecting concerning models too often (Kenny et al., 2015, p. 497; Taasoobshirazi & Wang, 2016, pp. 34, 36-37).

Latent state models

To compute the parcels' reliability, to determine spirituality's latent stability over time and to obtain latent mean differences in spirituality between first-time parents and controls for each wave, we estimated multigroup *latent state models* for every matched sample (Eid & Kutscher, 2014).
In a latent state model, one *latent state* is defined per measurement occasion and measured by all indicators available from the respective point in time (Eid & Kutscher, 2014, p. 265). Different measurement occasions' latent states can be correlated, allowing for statements about the stability of interindividual differences in latent states over time (ibid.). It has to be noted, however, that these stability coefficients do not represent how few the true scores of individuals change: They only indicate how few interindividual differences *in intraindividual change* occur, implying that if "all individuals change in the same way on the latent level, the latent retest correlation will be perfect" (ibid.).

Factor structure

In this study's multigroup latent state models, one latent spirituality factor was specified per wave (`S_w17` and `S_w20`), measured by the respective waves' three item parcels. Apart from one state spirituality factor per wave, indicator-specific factors were estimated to account for the parcels' semantic heterogeneity (Eid, 2000; Eid & Kutscher, 2014). The non-reference parcels 2 and 3 loaded on the two indicator-specific factors (`S2no` and `S3di`), each measured by the same non-reference parcel's values from both waves. These factors' correlations with the state factors were set to 0 (Eid & Kutscher, 2014, p. 266), while correlations between state factors as well as between indicator-specific factors were set free.

Consequently, the state factors represented the true score of the reference indicator and the indicator-specific factors represented their indicators' share over time that could not be predicted by the reference indicator (ibid.).

Strict measurement invariance

Strict measurement invariance assumptions were applied for all models to make latent factor means comparable across time and study groups (Eid & Kutscher, 2014, p. 267).
Note that each parcel's loadings and intercepts were set equal across study groups and waves, while the first parcel's loadings and all loadings on indicator-specific factors were fixed to 1, and all error variances were set equal across waves, but allowed to vary across study groups.

Scale of latent means

To define a reference point for comparisons between the latent means, the mean value of the baseline latent state factor was fixed to 0 in the CGs. The means of all three other latent state factors (the baseline factor in the TGs and the W20 factors in both groups) were set free.

The latent state models' specification is illustrated in Figure S3.

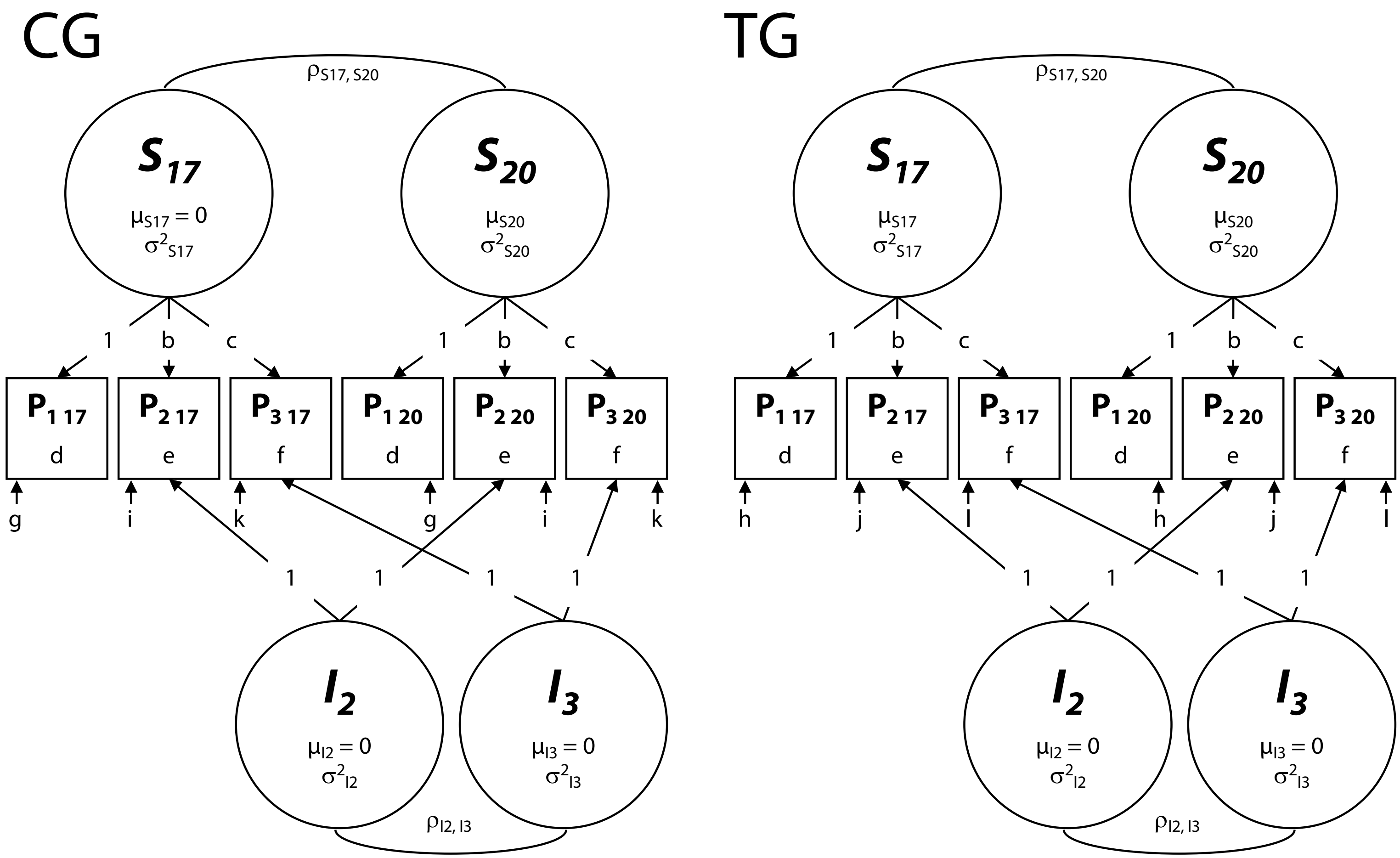

Figure S3: Latent state model. μ: Factor mean; σ^2: Factor variance; ρ: Factor covariance; 1, b and c: Factor loadings; d-f: Intercepts; g-l: Error variances. S17: Spirituality state factor at W17; S20: Spirituality state factor at W20; I2: Indicator-specific factor for parcel 2; I3: Indicator-specific factor for parcel 3; P1: Parcel 1 (general); P2: Parcel 2 (non-divine); P3: Parcel 3 (divine).

Latent change models

As second step, interested in differences between TG and CG members' latent spirituality change over time, we estimated multigroup latent *change* models for every matched sample to illustrate stability and change of spirituality in a different way. In latent change models, participants' values on the indicators are not specified as functions of a latent state at a given measurement occasion (Steyer et al., 1997, pp. 23-24). Rather, a general factor represents the variance that is common to all measurement occasions. In applications with data from two measurement occasions, a latent *change* factor then represents the amount of systematic variance in the second measurement occasion's indicators that cannot be explained by the general factor (ibid.). In short, latent change models attribute interindividual differences at a given measurement occasion to interindividual differences at baseline and interindividual differences in intraindividual change since baseline.

It can be shown that latent change models are data equivalent transformations of respective latent state models (p. 25). This is the case in the current study: As all other configurations remained unchanged, our latent change models were data equivalent to (i.e., yielded the same fit as) the latent state models.

Factor structure

In this study's latent change models, one latent baseline spirituality factor was specified (`S_w17`), measured by *all* waves' item parcels. To represent latent change, W20's three item parcels additionally loaded on a latent change factor (`S_change`).

As in the latent state models, the non-reference parcels 2 and 3 additionally loaded on two indicator-specific factors (`S2no` and `S3di`), each measured by the same non-reference parcel's values from both waves. These factors' correlations with the baseline spirituality and the spiritual change factors were set to 0, while correlations between spirituality factors (baseline and change) as well as between indicator-specific factors were set free.

Strict measurement invariance

As in the latent state models, strict measurement invariance assumptions were implemented setting each parcel's loadings equal across factors, study groups and waves, while the first parcel's loadings and all loadings on the indicator-specific factors were fixed to 1, setting each parcel's intercepts equal across study groups and waves and setting each parcel's error variances equal across waves, but allowing them to vary across study groups.

Scale of latent means

To define a reference point for comparisons between the latent means, the mean value of the latent baseline spirituality factor was fixed to 0 in the CGs. Its mean values in the TGs as well as the spiritual change factors' mean values in both groups were set free.

The latent change models' specification is illustrated in Figure S4.

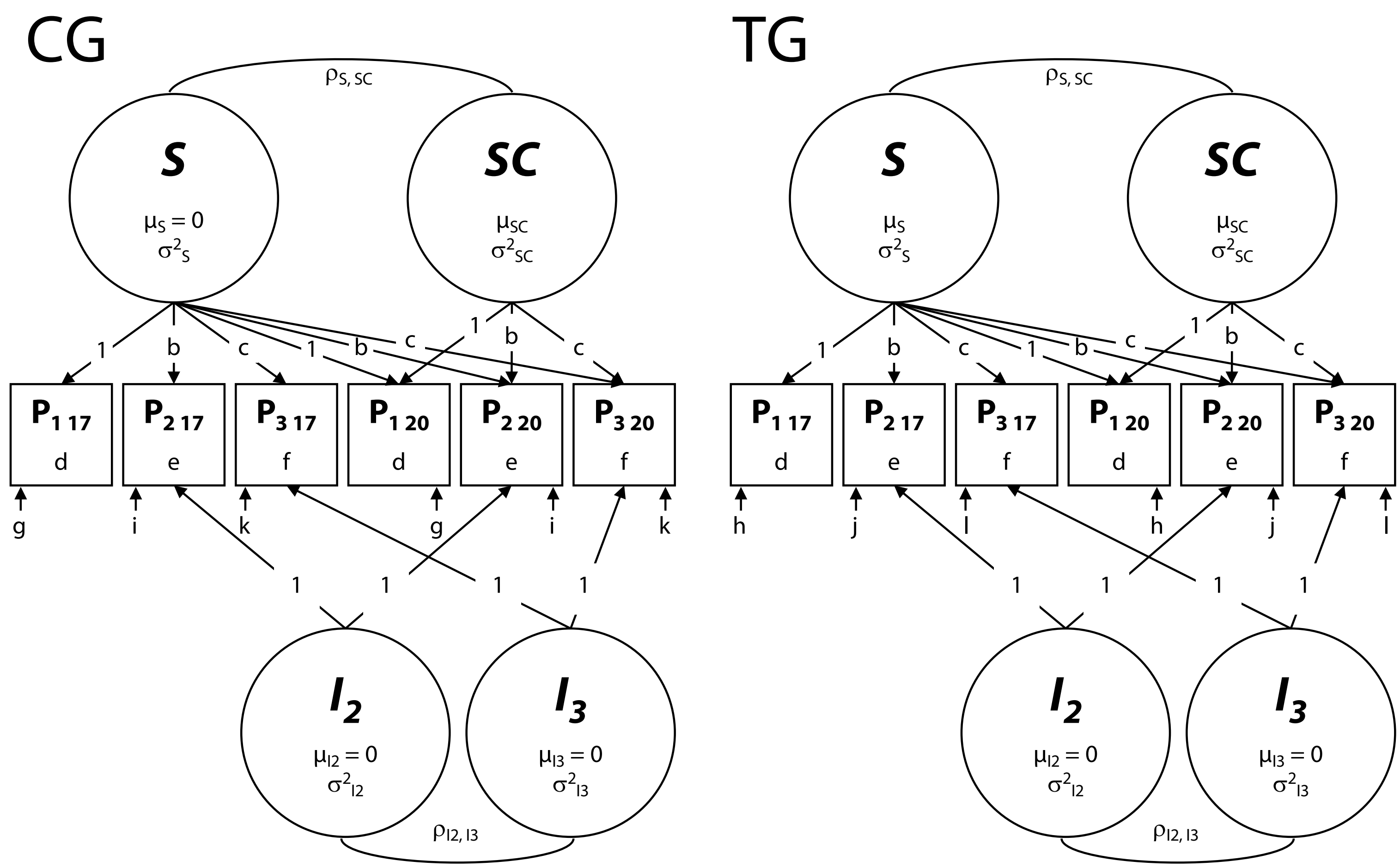

Figure S4: Latent change model. μ: Factor mean; σ^2: Factor variance; ρ: Factor covariance; 1, b and c: Factor loadings; d-f: Intercepts; g-l: Error variances. S: General spirituality factor; SC: Spiritual change factor; I2: Indicator-specific factor for parcel 2; I3: Indicator-specific factor for parcel 3; P1: Parcel 1 (general); P2: Parcel 2 (non-divine); P3: Parcel 3 (divine).

Comparison of study groups' latent change

The latent change models provide information on whether or not TG members' latent spirituality changed from pre- to post-birth (i.e., from W17 to W20) on average. However, as only the mean of CG members' general spirituality factor was set to 0 and the latent means of both groups' change factors were set free, a comparison between both groups' latent change means - essential for the purpose of the present study - required additional model constraints.

Models with additional restrictions

To evaluate whether first-time parents' spiritual change differed from controls', i.e., to be able to compare both groups' latent change means, two additional models were estimated for each sample. Both models were identical with the latent change models estimated earlier except for the following differences:

- In the first additional model which we call *comparison model*, the mean of the CG's first spirituality factor was set free and the mean of the CG's latent change factor was set to 0.
- In the second additional model which we call *test model*, the mean of the CG's first spirituality factor was set free and the means of both groups' latent change factors were set to 0.

Model comparison

To compare both models, a *Likelihood Ratio Test* was conducted in each sample. Whenever the test model did not fit the data significantly worse than the comparison model, we assumed that the respective TG's latent spirituality had not changed differently than the CG's latent spirituality.

Supplement 7: Results

Propensity score matching

Overall matching success

As shown in Table S4, 436 $< n_{\text{all}} <$ 969 individuals were considered for matching in the samples including first-time parents (TG) and childless controls (CG1) and 82 $< n_{\text{all}} <$ 131 in those containing parents who got a further child (CG2) as controls.

Some TG candidates had missings on basic covariates. The proportion of TG candidates represented (above 60% in 20 of 23 matched samples) may indicate how likely a given sample's results approached the ATT. Interestingly, covariate balance was often satisfactory despite unsatisfying PS overlap.

Table S4: Overview of matching success

Sample	Sel.[a]	n_{all}[b]	R^2_N[c]	$n_{matched}$[d]	$\%_{TG}$[e]	Covariate balance
F1	1	831	0.24	122	88.4	satisfactory except partnership/family
	2	772	0.369	120	87	satisfactory except partnership/family
	3	686	0.388	118	85.5	satisfactory
	4	831	0.281	122	88.4	satisfactory except partnership/family
	5	587	0.307	94	68.1	satisfactory except partnership/family
	6	460	0.626	90	65.2	satisfactory
M1	1	969	0.276	118	85.5	partially acceptable
	2	887	0.332	110	79.7	partially satisfactory
	3	636	0.359	112	81.2	acceptable
	4	968	0.301	118	85.5	partially acceptable
	5	680	0.366	96	69.6	satisfactory
	6	436	0.475	88	63.8	acceptable
F2	1	131	0.232	122	88.4	satisfactory except time-related
	2	120	0.56	92	66.7	satisfactory except time-related
	3	127	0.351	118	85.5	satisfactory except time-related
	4	131	0.301	122	88.4	satisfactory except time-related
	5	89	0.237	82	59.4	satisfactory except time-related
	6	82	0.54	64	46.4	acceptable except time-related
M2	1	125	0.228	116	84.1	satisfactory except time-related
	2	120	0.484	96	69.6	satisfactory except time-related
	3	114	0.574	96	69.6	satisfactory except time-related
	4	125	0.471	94	68.1	acceptable except time-related
	6	109	0.79	62	44.9	acceptable except time-related

Note. [a]Matching variable selection. [b]Number of TG and CG1 or TG and CG2 candidates without missing values on the covariates used for matching in the given selection. [c]Nagelkerke's effect size yielded in the logistic regression model used for propensity score estimation. [d]Size of the matched sample. [e]Proportion of all 69 female/male TG candidates (all, i.e., not only those without missing values on the covariates used) in the matched sample.

Matched sample characteristics

As shown in Figure S5, matched samples' TGs and CG2s largely consisted of the same individuals, while most CG1 members were only matched to one sample.

On average, matched individuals were around 30 years old ($29.08 \leq M_{\text{Age}} \leq 34.55$; $4.39 \leq SD_{\text{Age}} \leq 9.82$). TG/CG2 samples tended to be slightly older, as were male samples, where age was also occasionally less homogeneous. The vast majority of participants had a Swiss first nationality ($89.83 \leq \%_{\text{Swiss}} \leq 97.27$), lived in a relationship ($71.28 \leq \%_{\text{Partner}} \leq 100$, high proportions especially frequent in TG/CG2 samples) and indicated having a religious denomination ($77.12 \leq \%_{\text{Denomination}} \leq 90.62$). Manifest baseline spirituality was below the centre of the scale in all samples ($6.38 \leq M_{\text{Baseline}} \leq 8.54$) and usually slightly higher in women (see Table S5).

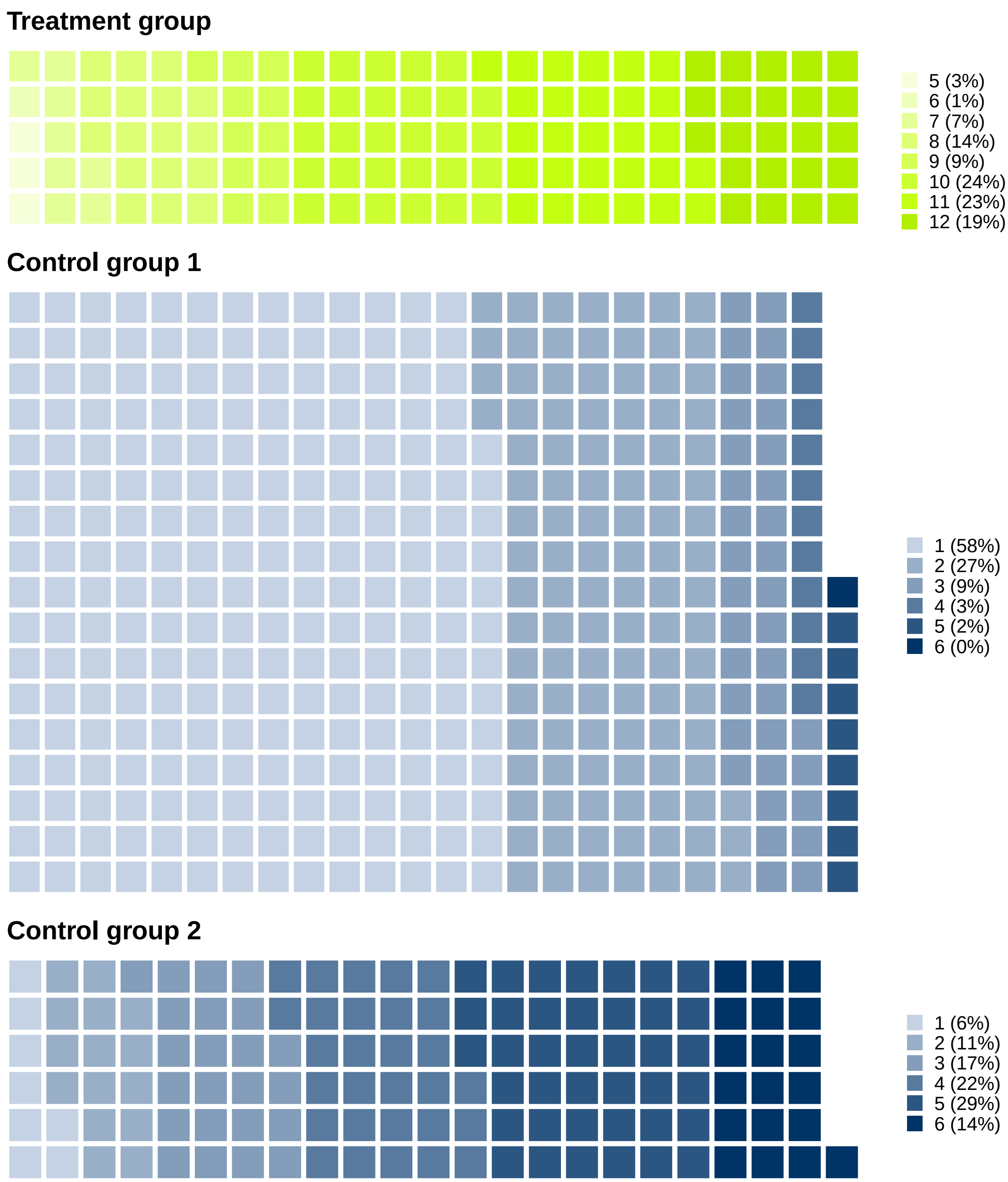

Figure S5: Number of matched samples that SHP participants matched at least once were matched to

Table S5: Central characteristics of the matched samples

**Sample**	**Group**	**n**	**Age**	**Swiss**[a]	**Partner**[b]	**Denom.**[c]	**Spirituality**[d]
			M (SD)	%	%	%	M (SD)
F1M1	TG	61	29.20 (5.32)	93.44	98.36	88.52	7.92 (3.73)
	CG	61	29.54 (5.68)	93.44	63.93	73.77	7.93 (3.34)
F1M2	TG	60	29.30 (5.31)	93.33	98.33	88.33	7.95 (3.75)
	CG	60	30.12 (6.34)	96.67	61.67	86.67	8.04 (4.67)
F1M3	TG	59	29.56 (5.01)	93.22	100.00	88.14	8.02 (3.75)
	CG	59	30.73 (5.69)	86.44	89.83	76.27	8.04 (3.64)
F1M4	TG	61	29.20 (5.32)	93.44	98.36	88.52	7.92 (3.73)
	CG	61	29.30 (5.54)	88.52	52.46	78.69	7.81 (4.67)
F1M5	TG	47	28.98 (5.03)	93.62	97.87	87.23	8.40 (3.87)
	CG	47	29.17 (5.29)	93.62	44.68	78.72	8.32 (4.60)
F1M6	TG	45	29.38 (4.72)	93.33	100.00	86.67	8.51 (3.92)
	CG	45	29.56 (5.94)	95.56	75.56	80.00	7.76 (3.50)
M1M1	TG	59	32.46 (8.67)	94.92	89.83	83.05	6.91 (4.38)
	CG	59	32.64 (8.13)	86.44	62.71	71.19	6.63 (3.82)
M1M2	TG	55	32.31 (8.93)	94.55	90.91	81.82	7.01 (4.46)
	CG	55	31.64 (5.75)	100.00	63.64	87.27	6.60 (3.90)
M1M3	TG	56	31.21 (4.85)	94.64	89.29	83.93	6.96 (4.47)
	CG	56	32.59 (5.42)	91.07	91.07	80.36	6.83 (4.20)
M1M4	TG	59	32.46 (8.67)	94.92	89.83	83.05	6.91 (4.38)
	CG	59	33.24 (10.92)	91.53	62.71	79.66	7.34 (4.65)
M1M5	TG	48	31.04 (4.97)	97.92	89.58	85.42	7.25 (4.74)
	CG	48	31.75 (4.36)	93.75	70.83	72.92	6.32 (3.78)
M1M6	TG	44	30.82 (4.94)	97.73	90.91	84.09	7.41 (4.85)
	CG	44	32.23 (6.10)	95.45	93.18	84.09	7.35 (4.08)
F2M1	TG	61	29.20 (5.32)	93.44	98.36	88.52	7.92 (3.73)
	CG	61	32.02 (3.76)	86.89	98.36	90.16	7.98 (4.43)
F2M2	TG	46	29.22 (5.64)	95.65	97.83	89.13	8.28 (3.79)
	CG	46	32.20 (3.58)	89.13	97.83	89.13	7.38 (4.57)
F2M3	TG	59	29.56 (5.01)	93.22	100.00	88.14	8.02 (3.75)
	CG	59	32.46 (3.80)	86.44	98.31	91.53	7.57 (4.40)
F2M4	TG	61	29.20 (5.32)	93.44	98.36	88.52	7.92 (3.73)
	CG	61	32.23 (3.84)	91.80	96.72	90.16	8.12 (4.25)

Sample	Group	n	Age	Swiss[a]	Partner[b]	Denom.[c]	Spirituality[d]
			M (SD)	%	%	%	M (SD)
F2M5	TG	41	28.90 (5.30)	92.68	97.56	87.80	8.63 (4.00)
	CG	41	31.98 (3.76)	97.56	100.00	90.24	7.61 (4.26)
F2M6	TG	32	29.34 (5.12)	90.62	100.00	90.62	8.82 (4.24)
	CG	32	31.94 (3.65)	100.00	100.00	90.62	8.25 (4.23)
M2M1	TG	58	32.48 (8.74)	94.83	89.66	82.76	6.82 (4.36)
	CG	58	36.62 (10.05)	86.21	96.55	87.93	6.41 (3.93)
M2M2	TG	48	31.77 (8.15)	93.75	89.58	83.33	7.40 (4.60)
	CG	48	37.17 (11.48)	91.67	95.83	87.50	6.88 (4.39)
M2M3	TG	48	31.19 (4.68)	93.75	89.58	83.33	6.62 (4.21)
	CG	48	33.92 (4.30)	91.67	100.00	89.58	6.22 (3.84)
M2M4	TG	47	32.62 (9.40)	95.74	89.36	78.72	6.50 (4.27)
	CG	47	34.09 (5.59)	91.49	100.00	89.36	6.70 (3.98)
M2M6	TG	31	30.42 (4.75)	93.55	87.10	80.65	6.92 (4.61)
	CG	31	33.23 (4.37)	93.55	100.00	87.10	5.84 (3.86)

Note. [a]Percentage of participants whose first nationality was Swiss; [b]Percentage of participants who lived in a relationship; [c]Percentage of individuals who reported a denomination; [d]Manifest mean of the seven spirituality items at W17.

Manifest matching-based analyses

t-tests for independent samples

All samples fulfilled the preconditions described in Supplement 5 (see code file).

Manifest retest correlations

Table S6 shows the matched samples' manifest retest correlations of spirituality scores.

Table S6: Manifest retest reliabilities of spirituality scores

Sample[a]	Sel.[b]	Group	r [95% CI][c]	t	df	p
F1	1	TG	0.87 [0.79;0.92]	12.66	51	0
		CG	0.68 [0.52;0.80]	7.08	57	0
	2	TG	0.87 [0.78;0.92]	12.55	50	0
		CG	0.89 [0.81;0.93]	14.27	56	0
	3	TG	0.87 [0.78;0.92]	12.25	49	0
		CG	0.85 [0.76;0.91]	11.83	54	0
	4	TG	0.87 [0.79;0.92]	12.66	51	0
		CG	0.90 [0.84;0.94]	15.70	55	0
	5	TG	0.86 [0.75;0.92]	10.44	38	0
		CG	0.88 [0.78;0.93]	11.80	42	0
	6	TG	0.86 [0.74;0.92]	10.08	36	0
		CG	0.83 [0.71;0.90]	9.66	42	0
F2	1	TG	0.87 [0.79;0.92]	12.66	51	0
		CG	0.85 [0.76;0.91]	11.64	50	0
	2	TG	0.88 [0.79;0.94]	11.43	37	0
		CG	0.93 [0.87;0.96]	15.63	37	0
	3	TG	0.87 [0.78;0.92]	12.25	49	0
		CG	0.93 [0.87;0.96]	17.37	50	0
	4	TG	0.87 [0.79;0.92]	12.66	51	0
		CG	0.83 [0.73;0.90]	10.70	50	0
	5	TG	0.86 [0.74;0.93]	9.52	32	0
		CG	0.83 [0.69;0.91]	8.82	35	0
	6	TG	0.87 [0.73;0.94]	8.92	26	0
		CG	0.92 [0.84;0.96]	12.35	27	0

Sample[a]	Sel.[b]	Group	r [95% CI][c]	t	df	p
M1	1	TG	0.92 [0.86;0.95]	16.47	52	0
		CG	0.80 [0.69;0.88]	9.98	55	0
	2	TG	0.91 [0.85;0.95]	15.90	50	0
		CG	0.77 [0.62;0.86]	8.17	47	0
	3	TG	0.92 [0.87;0.96]	16.71	49	0
		CG	0.87 [0.77;0.92]	12.07	49	0
	4	TG	0.92 [0.86;0.95]	16.47	52	0
		CG	0.86 [0.78;0.92]	12.66	55	0
	5	TG	0.92 [0.86;0.96]	15.55	43	0
		CG	0.91 [0.85;0.95]	14.69	43	0
	6	TG	0.92 [0.85;0.96]	14.78	40	0
		CG	0.92 [0.85;0.95]	14.29	39	0
M2	1	TG	0.91 [0.85;0.95]	16.04	51	0
		CG	0.92 [0.87;0.96]	16.63	48	0
	2	TG	0.95 [0.90;0.97]	18.95	43	0
		CG	0.94 [0.90;0.97]	17.86	39	0
	3	TG	0.90 [0.83;0.94]	13.62	43	0
		CG	0.92 [0.85;0.95]	14.71	41	0
	4	TG	0.91 [0.84;0.95]	13.87	40	0
		CG	0.92 [0.85;0.96]	14.24	38	0
	6	TG	0.92 [0.84;0.96]	12.30	27	0
		CG	0.92 [0.82;0.96]	11.47	25	0

Note. [a]Sample; [b]Matching variable selection; [c]Manifest retest correlation of spirituality scores at W17 and W20 including a 95% confidence interval.

Latent matching-based analyses

Latent state models

Model fit. Latent state models' fit characteristics for the 23 samples are shown in Table S7. 19 models showed non-significant χ^2 values, the other four models had *p*-values of .003, .041, .045 and .01. All models' χ^2 values were lower than twice the degrees of freedom. 22 CFI values were larger than .95, 17 larger than .97, the RMSEA values of 14 models were smaller than .05, 21 RMSEA confidence intervals covered .05, the largest RMSEA value was 0.153. In general, the model fit was very good to acceptable.

Factor loadings. Across samples, study groups and waves, standardised state factor loadings were high for the first (general) and the third ('divine'), but smaller for the second ('non-divine') parcel (see Table S8). Interestingly, this difference was weaker in men, especially first-time fathers, suggesting that latent spirituality represented non-religious experiences and behaviours to a greater extent in this group.

Latent reliabilities. Likewise, the first and third parcels showed high ($M_{\text{Rel. 1}} = .78$) and very high ($M_{\text{Rel. 3}} = .93$) latent reliabilities, while the second parcel's reliabilities were only high in the male samples ($M_{\text{Rel. 2}♂} = 0.71$, $M_{\text{Rel. 2}♀} = 0.54$; see Table S9). This suggest that 'non-divine' experiences/behaviours contributed less to measuring spirituality in women.

Conclusion. Given the very good to acceptable model fit and the sufficient to high parcel reliabilities in all samples, it can be proceeded on the strict measurement invariance assumptions and presumed spirituality was reliably measured in the same way across groups and waves.

Latent change models

Factor loadings. The same pattern as for state factor loadings in the latent state models was observed regarding spiritual change in the latent change models, but loadings were generally much smaller here (see Table S10).

Central findings from latent state and latent change models

Latent stability and change. Stability and change coefficients obtained from the latent state and latent change models are illustrated in Table S11. Table S12 shows the results of the Likelihood Ratio Tests comparing a multigroup latent change model per sample in which the means of *both* groups' latent change factors were set to zero to a model in which the *TG's* mean was set free. Note than in three of the latent models, lavaan estimated latent stabilities > 1 and, consequently, negative latent change factor variances. Affected models are marked in the tables.

Table S7: Model fit characteristics of latent state (and change) models

Sample Sel.[a]		n	χ^2	df	p	CFI[b]	RMSEA[c] [95% CI]	SRMR[d]
F1	1	122	29.32	28	.396	.996	.010, [.000;.107]	.092
	2	120	24.20	28	.671	1.000	.000, [.000;.085]	.069
	3	118	24.96	28	.630	1.000	.000, [.000;.089]	.084
	4	122	26.91	28	.523	1.000	.000, [.000;.099]	.082
	5	94	39.81	28	.069	.956	.095, [.000;.165]	.112
	6	90	23.08	28	.729	1.000	.000, [.000;.083]	.088
F2	1	122	35.80	28	.148	.977	.065, [.000;.134]	.076
	2	92	39.63	28	.071	.963	.110, [.000;.181]	.089
	3	118	24.44	28	.658	1.000	.000, [.000;.090]	.078
	4	122	29.89	28	.369	.994	.018, [.000;.114]	.076
	5	82	22.92	28	.737	1.000	.000, [.000;.093]	.104
	6	64	23.58	28	.703	1.000	.000, [.000;.114]	.111
M1	1	118	40.42	28	.061	.973	.085, [.000;.145]	.137
	2	110	29.66	28	.380	.996	.029, [.000;.116]	.089
	3	112	27.53	28	.490	1.000	.000, [.000;.110]	.061
	4	118	29.98	28	.364	.996	.035, [.000;.111]	.081
	5	96	24.30	28	.666	1.000	.000, [.000;.092]	.075
	6	88	29.16	28	.404	.997	.022, [.000;.123]	.082
M2	1	116	52.49	28	.003	.955	.126, [.072;.179]	.082
	2	96	42.24	28	.041	.970	.106, [.026;.168]	.107
	3	96	41.86	28	.045	.968	.102, [.011;.163]	.082
	4	94	40.97	28	.054	.969	.098, [.000;.163]	.085
	6	62	48.44	28	.010	.936	.153, [.071;.227]	.092

Note. The estimated latent state and change models were data equivalent and yielded the same fit. [a]Matching variable selection; [b]Comparative Fit Index; [c]Root Mean Square Error of Approximation; [d]Standardised Root Mean Square Residual.

Table S8: Factor loadings in the latent state models

**Sample**	**Sel.**[a]	**S_{17}**[b]						**S_{20}**[c]					
		W17[d]						W20[e]					
		TG			CG			TG			CG		
		P1[f]	P2[g]	P3[h]	P1[f]	P2[g]	P3[h]	P1[f]	P2[g]	P3[h]	P1[f]	P2[g]	P3[h]
F1	1	0.83	0.42	0.81	0.86	0.35	0.77	0.87	0.47	0.85	0.87	0.36	0.78
	2	0.82	0.43	0.83	0.91	0.49	0.89	0.86	0.48	0.86	0.91	0.49	0.89
	3	0.83	0.38	0.82	0.87	0.37	0.81	0.86	0.42	0.86	0.88	0.40	0.83
	4	0.83	0.44	0.82	0.90	0.47	0.86	0.86	0.50	0.86	0.90	0.46	0.85
	5	0.80	0.44	0.85	0.82	0.46	0.92	0.83	0.47	0.86	0.84	0.48	0.93
	6	0.79	0.35	0.88	0.76	0.27	0.80	0.81	0.37	0.89	0.79	0.30	0.82
F2	1	0.83	0.46	0.81	0.94	0.55	0.83	0.87	0.52	0.85	0.95	0.55	0.83
	2	0.85	0.44	0.82	0.92	0.57	0.88	0.87	0.47	0.84	0.92	0.55	0.87
	3	0.83	0.44	0.82	0.92	0.54	0.81	0.86	0.48	0.85	0.91	0.53	0.80
	4	0.83	0.44	0.82	0.92	0.47	0.80	0.87	0.49	0.85	0.93	0.49	0.81
	5	0.83	0.46	0.79	0.93	0.51	0.76	0.85	0.50	0.82	0.92	0.50	0.76
	6	0.82	0.47	0.79	0.93	0.47	0.73	0.84	0.50	0.82	0.93	0.47	0.73
M1	1	0.90	0.56	0.76	0.93	0.47	0.78	0.90	0.56	0.77	0.92	0.46	0.77
	2	0.90	0.65	0.77	0.93	0.59	0.73	0.91	0.65	0.78	0.90	0.51	0.65
	3	0.91	0.66	0.78	0.92	0.60	0.71	0.91	0.66	0.78	0.90	0.55	0.67
	4	0.89	0.62	0.80	0.97	0.63	0.80	0.89	0.62	0.80	0.96	0.60	0.78
	5	0.91	0.70	0.80	0.83	0.53	0.70	0.91	0.70	0.80	0.85	0.56	0.73
	6	0.92	0.73	0.79	0.92	0.61	0.71	0.92	0.73	0.78	0.92	0.61	0.72
M2	1	0.87	0.64	0.84	0.87	0.54	0.77	0.87	0.64	0.84	0.87	0.54	0.76
	2	0.88	0.70	0.85	0.88	0.57	0.82	0.89	0.70	0.85	0.89	0.58	0.83
	3	0.88	0.70	0.77	0.88	0.64	0.70	0.88	0.70	0.77	0.87	0.63	0.69
	4	0.88	0.64	0.81	0.85	0.55	0.72	0.89	0.65	0.82	0.84	0.54	0.71
	6	0.93	0.69	0.82	0.93	0.63	0.76	0.93	0.70	0.83	0.91	0.59	0.72

Note. [a]Matching variable selection; [b]Standardised factor loadings on the spirituality state factor assigned to W17; [c]Standardised factor loadings on the spirituality state factor assigned to W20; [d]Standardised factor loadings of parcels containing items measured at W17; [e]Standardised factor loadings of parcels containing items measured at W20; [f]Parcel 1 (general parcel; mean of the items *Feeling of spirituality* and *Think about religious (and spiritual) questions*); [g]Parcel 2 (non-divine parcel; mean of the items *Feeling to be at one* and *Meditation (frequency)*); [h]Parcel 3 (divine parcel; mean of the items *Belief in God or something divine*, *Feeling that God (or something divine) intervenes in life* and *Prayer (frequency))*.

Table S9: Latent parcel reliabilities in latent state (and change) models

**Sample Sel.**[a]		**P1**[b]				**P2**[c]				**P3**[d]			
		W17		W20		W17		W20		W17		W20	
		TG	CG	TG	CG	TG	CG	TG	CG	TG	CG	TG	CG
F1	1	.70	.73	.75	.75	.54	.51	.57	.52	.92	.99	.94	.99
	2	.67	.83	.74	.83	.54	.53	.56	.53	.93	.95	.94	.95
	3	.69	.75	.74	.78	.52	.56	.54	.57	.93	.98	.94	.98
	4	.68	.81	.74	.81	.55	.61	.58	.61	.93	.94	.94	.94
	5	.65	.67	.68	.70	.51	.61	.52	.62	.95	.95	.96	.96
	6	.63	.58	.66	.63	.45	.62	.46	.63	.96	.96	.96	.97
F2	1	.69	.89	.75	.89	.55	.46	.58	.47	.92	.91	.94	.91
	2	.72	.85	.75	.84	.62	.56	.63	.55	.95	.90	.96	.89
	3	.68	.84	.74	.83	.54	.58	.56	.57	.93	.92	.94	.92
	4	.69	.85	.75	.86	.54	.44	.57	.45	.92	.91	.94	.91
	5	.68	.86	.73	.85	.50	.46	.52	.45	.94	.92	.94	.92
	6	.67	.86	.71	.86	.41	.53	.43	.53	.93	.91	.94	.91
M1	1	.81	.86	.82	.86	.70	.61	.70	.60	.94	.86	.94	.85
	2	.82	.87	.82	.81	.75	.65	.75	.61	.94	.82	.94	.78
	3	.83	.84	.83	.81	.72	.77	.72	.76	.93	.89	.93	.88
	4	.79	.93	.80	.92	.71	.76	.71	.74	.95	.89	.95	.88
	5	.83	.70	.83	.73	.69	.83	.69	.83	.95	.87	.95	.88
	6	.85	.84	.84	.85	.75	.77	.74	.77	.95	.90	.94	.90
M2	1	.76	.76	.76	.76	.71	.67	.71	.66	.96	.96	.96	.95
	2	.78	.78	.78	.78	.77	.62	.77	.63	.95	.95	.95	.95
	3	.77	.78	.77	.76	.72	.63	.72	.62	.93	.95	.93	.95
	4	.77	.73	.78	.71	.63	.67	.64	.66	.96	.95	.96	.95
	6	.86	.86	.86	.83	.82	.70	.83	.67	.95	.92	.95	.91

Note. [a]Matching variable selection; [b]Parcel 1 (general parcel; mean of the items *Feeling of spirituality* and *Think about religious (and spiritual) questions*); [c]Parcel 2 (non-divine parcel; mean of the items *Feeling to be at one* and *Meditation (frequency)*); [d]Parcel 3 (divine parcel; mean of the items *Belief in God or something divine*, *Feeling that God (or something divine) intervenes in life* and *Prayer (frequency)*).

Table S10: Factor loadings in the latent change models

**S.**[a]	**Spirituality**[b]												**Spiritual Change**[c]					
	TG						CG						TG			CG		
	W17[d]			W20[e]			W17[d]			W20[e]			W20[e]			W20[e]		
	P1[f]	P2[g]	P3[h]	P1[f]	P2[g]	P3[h]	P1[f]	P2[g]	P3[h]	P1[f]	P2[g]	P3[h]	P1[f]	P2[g]	P3[h]	P1[f]	P2[g]	P3[h]
F1																		
1	0.83	0.42	0.81	0.75	0.40	0.73	0.86	0.35	0.77	0.83	0.35	0.75	0.27	0.14	0.26	0.57	0.24	0.52
2	0.82	0.43	0.83	0.74	0.41	0.75	0.91	0.49	0.89	0.91	0.49	0.88	0.26	0.15	0.27	0.27	0.15	0.27
3	0.83	0.38	0.82	0.76	0.37	0.75	0.87	0.37	0.81	0.82	0.37	0.77	0.26	0.13	0.26	0.34	0.15	0.32
4	0.83	0.44	0.82	0.74	0.43	0.74	0.90	0.47	0.86	0.91	0.47	0.86	0.27	0.15	0.26	0.15	0.08	0.14
5	0.80	0.44	0.85	0.76	0.44	0.80	0.82	0.46	0.92	0.79	0.45	0.87	0.25	0.15	0.27	0.25	0.14	0.27
6	0.79	0.35	0.88	0.76	0.35	0.84	0.76	0.27	0.80	0.72	0.27	0.75	0.25	0.11	0.27	0.28	0.11	0.29
F2																		
1	0.83	0.46	0.81	0.74	0.44	0.73	0.94	0.55	0.83	0.93	0.54	0.82	0.27	0.16	0.26	0.35	0.20	0.31
2	0.85	0.44	0.82	0.80	0.43	0.78	0.92	0.57	0.88	0.95	0.57	0.90	0.25	0.14	0.24	0.14	0.08	0.13
3	0.83	0.44	0.82	0.75	0.43	0.74	0.92	0.54	0.81	0.95	0.55	0.83	0.26	0.15	0.26	0.11	0.06	0.10
4	0.83	0.44	0.82	0.75	0.43	0.73	0.92	0.47	0.80	0.89	0.47	0.78	0.27	0.15	0.26	0.34	0.18	0.29
5	0.83	0.46	0.79	0.77	0.45	0.74	0.93	0.51	0.76	0.94	0.51	0.77	0.26	0.15	0.25	0.38	0.20	0.31
6[i]	*0.82*	*0.47*	*0.79*	*0.77*	*0.46*	*0.75*	*0.93*	*0.47*	*0.73*	*0.92*	*0.47*	*0.72*	*0.22*	*0.13*	*0.22*	*NA*[i]	*NA*[i]	*NA*[i]
M1																		
1	0.90	0.56	0.76	0.89	0.55	0.76	0.93	0.47	0.78	0.95	0.47	0.79	0.29	0.18	0.25	0.33	0.17	0.28
2	0.90	0.65	0.77	0.90	0.65	0.77	0.93	0.59	0.73	1.12	0.63	0.80	0.28	0.20	0.24	0.48	0.27	0.35
3	0.91	0.66	0.78	0.91	0.66	0.78	0.92	0.60	0.71	1.00	0.62	0.75	0.26	0.18	0.22	0.35	0.22	0.26
4	0.89	0.62	0.80	0.88	0.61	0.79	0.97	0.63	0.80	1.03	0.65	0.84	0.27	0.19	0.24	0.37	0.23	0.30
5[i]	*0.91*	*0.70*	*0.80*	*0.91*	*0.70*	*0.80*	*0.83*	*0.53*	*0.70*	*0.79*	*0.52*	*0.68*	*0.26*	*0.20*	*0.23*	*NA*[i]	*NA*[i]	*NA*[i]
6	0.92	0.73	0.79	0.93	0.73	0.79	0.92	0.61	0.71	0.90	0.60	0.70	0.28	0.22	0.24	0.14	0.09	0.11
M2																		
1	0.87	0.64	0.84	0.87	0.63	0.84	0.87	0.54	0.77	0.88	0.55	0.77	0.28	0.20	0.27	0.12	0.07	0.10
2[i]	*0.88*	*0.70*	*0.85*	*0.88*	*0.69*	*0.84*	*0.88*	*0.57*	*0.82*	*0.87*	*0.57*	*0.81*	*0.09*	*0.07*	*0.09*	*NA*[i]	*NA*[i]	*NA*[i]
3	0.88	0.70	0.77	0.88	0.70	0.77	0.88	0.64	0.70	0.90	0.65	0.71	0.27	0.22	0.24	0.18	0.13	0.14
4	0.88	0.64	0.81	0.85	0.63	0.79	0.85	0.55	0.72	0.88	0.56	0.74	0.29	0.21	0.27	0.11	0.07	0.09
6	0.93	0.69	0.82	0.90	0.68	0.81	0.93	0.63	0.76	1.02	0.66	0.80	0.29	0.22	0.26	0.23	0.14	0.18

Note. [a]Sample and matching variable selection; [b]Standardised factor loadings on the general spirituality factor; [c]Standardised factor loadings on the spiritual change factor; [d]Standardised factor loadings of parcels containing items measured at W17; [e]Standardised factor loadings of parcels containing items measured at W20; [f]Parcel 1 (general parcel; mean of the items *Feeling of spirituality* and *Think about religious (and spiritual) questions*); [g]Parcel 2 (non-divine parcel; mean of the items *Feeling to be at one* and *Meditation (frequency)*); [h]Parcel 3 (divine parcel; mean of the items *Belief in God or something divine*, *Feeling that God (or something divine) intervenes in life* and *Prayer (frequency));* [i]In the latent state models corresponding to the three models printed in italic, latent stabilities > 1 were estimated. Consequently, lavaan estimated negative variances of the latent change factor, which is why no standardised factor loadings could be estimated for these factors.

Table S11: Latent stability and change of spirituality

**S.**[a]	**Stability**[b]		**Change**[c]								
	TG	CG	TG				CG				Diff.[d]
			Est.[e]	SE[f]	z	p	Est.[e]	SE[f]	z	p	Est.[e]
F1											
1	.96	.77	-0.188	0.297	-0.633	.527	-0.089	0.327	-0.271	.786	-0.099
2	.96	.96	-0.181	0.286	-0.634	.526	-0.448	0.283	-1.585	.113	0.267
3	.96	.92	-0.084	0.292	-0.289	.773	-0.377	0.255	-1.477	.140	0.293
4	.96	.99	-0.182	0.288	-0.632	.528	-0.305	0.234	-1.306	.192	0.123
5	.95	.96	0.012	0.286	0.042	.966	-0.301	0.274	-1.098	.272	0.313
6	.95	.94	0.081	0.271	0.301	.764	-0.421	0.229	-1.837	.066	0.502
F2											
1	.96	.93	-0.191	0.295	-0.646	.518	-0.32	0.337	-0.95	.342	0.129
2	.96	.99	-0.329	0.297	-1.11	.267	-0.623	0.311	-2	.045	0.293
3	.96	.99	-0.086	0.295	-0.294	.769	-0.575	0.245	-2.341	.019	0.488
4	.96	.93	-0.187	0.292	-0.639	.523	-0.514	0.323	-1.591	.112	0.327
5	.95	.92	0.082	0.379	0.216	.829	-0.423	0.411	-1.03	.303	0.505
6[g]	*.97*	*1.01*	*0.279*	*0.413*	*0.675*	*.500*	*-0.434*	*0.27*	*-1.609*	*.108*	*0.713*
M1											
1	.95	.94	0.196	0.319	0.616	.538	0.088	0.318	0.276	.783	0.109
2	.95	.91	0.156	0.322	0.486	.627	-0.496	0.375	-1.32	.187	0.652
3	.96	.94	0.092	0.31	0.296	.767	-0.365	0.363	-1.006	.315	0.457
4	.95	.93	0.203	0.295	0.686	.493	-0.478	0.324	-1.475	.140	0.68
5[g]	*.96*	*1.01*	*0.112*	*0.322*	*0.346*	*.729*	*-0.377*	*0.259*	*-1.454*	*.146*	*0.488*
6	.96	.99	0.09	0.351	0.256	.798	0.178	0.297	0.601	.548	-0.088
M2											
1	.95	.99	0.187	0.27	0.691	.490	-0.628	0.193	-3.251	.001	0.815
2[g]	*.99*	*1.01*	*-0.203*	*0.254*	*-0.799*	*.425*	*-0.783*	*0.197*	*-3.978*	*.000*	*0.58*
3	.95	.98	0.119	0.325	0.367	.713	-0.769	0.223	-3.454	.001	0.888
4	.95	.99	0.356	0.302	1.18	.238	-0.827	0.219	-3.772	.000	1.184
6	.95	.98	0.403	0.487	0.827	.409	-0.86	0.321	-2.682	.007	1.263

Note. [a]Sample; [b]Correlations between the two spirituality state factors in the latent state models; [c]Means of the spiritual change factors in the latent change models; [d]Differences of the spiritual change factors' means between TG and CG; [e]Estimate; [f]Standard error; [g]Note that for the three models printed in italic, stability coefficients >1 were estimated.

Table S12: Comparison of study groups' mean latent spiritual change

**Sample**	**Sel.**[a]	**Comparison model**[b]		**Test model**[c]		**LRT**[d]		
		χ^2	df	χ^2	df	$\Delta_{\chi 2}$	Δ_{df}	p
F1	1	31.05	28	31.47	29	0.32	1	.572
	2	28.82	28	29.22	29	0.32	1	.574
	3	29.38	28	29.47	29	0.07	1	.785
	4	31.13	28	31.53	29	0.32	1	.573
	5	44.25	28	44.25	29	0.00	1	.967
	6	26.05	28	26.14	29	0.10	1	.747
F2	1	39.48	28	39.90	29	0.33	1	.564
	2	49.23	28	50.54	29	1.05	1	.306
	3	32.60	28	32.69	29	0.08	1	.779
	4	35.58	28	36.00	29	0.33	1	.568
	5	25.90	28	25.95	29	0.05	1	.825
	6[e]	*26.89*	*28*	*27.32*	*29*	*0.60*	*1*	*.439*
M1	1	40.25	28	40.64	29	0.44	1	.506
	2	31.61	28	31.85	29	0.27	1	.603
	3	30.24	28	30.33	29	0.10	1	.756
	4	31.35	28	31.85	29	0.57	1	.452
	5[e]	*25.20*	*28*	*25.32*	*29*	*0.13*	*1*	*.719*
	6	29.94	28	30.00	29	0.07	1	.787
M2	1	58.17	28	58.64	29	0.52	1	.470
	2[e]	*53.38*	*28*	*54.12*	*29*	*0.52*	*1*	*.471*
	3	49.29	28	49.40	29	0.11	1	.735
	4	49.23	28	50.64	29	1.98	1	.160
	6	51.51	28	52.17	29	0.79	1	.376

Note. [a]Matching variable selection; [b]Latent change model in which the mean of the *Spiritual Change* factor was fixed to *0* in the CG; [c]Latent change model in which the means of the *Spiritual Change* factors were fixed to *0* in both groups; [d]Likelihood Ratio Test comparing both models; [e]Note that for the three models printed in italic, stability coefficients >1 and consequently negative change factor variances were estimated.

In line with the results on latent stability, the latent spiritual change means did not differ from 0 (i.e., the CGs' general spirituality mean) in first-time parents and most CGs ($p_{\mu SC} > .05$), neither did latent change variance. Latent spirituality slightly declined in two female and all male CG2 samples ($M_{\mu SC} = -0.72$; $p < .05$), however, there were no significant differences in latent change between groups in any sample ($0 \leq \Delta_{\chi^2} \leq 1.98$, $p_{\text{LRT}} > .05$).

State factor variances. Table S13 and Figure S6 show the latent state factor variances referred to in the manuscript.

Table S13: Spirituality state factor variances in the latent state models

Sample	Sel.[a]	TG			CG		
		S_{17}[b]	S_{20}[c]	Δ_{TG}[d]	S_{17}[b]	S_{20}[c]	Δ_{CG}[d]
F1	1	14.75	19.86	5.11	12.07	13.22	1.14
	2	14.21	19.04	4.84	22.34	22.49	0.15
	3	15.02	19.30	4.28	14.97	17.43	2.46
	4	14.32	19.28	4.96	22.07	21.48	-0.59
	5	14.08	16.54	2.46	18.18	20.66	2.48
	6	13.97	15.70	1.73	8.77	10.64	1.88
F2	1	14.54	19.74	5.20	20.42	21.07	0.66
	2	14.54	16.93	2.39	20.49	18.85	-1.63
	3	14.75	19.15	4.39	19.95	18.55	-1.40
	4	14.59	19.68	5.09	18.18	19.70	1.52
	5	17.15	20.92	3.77	18.93	18.27	-0.66
	6	19.23	22.85	3.62	18.77	19.34	0.57
M1	1	21.36	21.99	0.63	19.53	18.57	-0.96
	2	22.18	22.47	0.30	19.15	12.53	-6.62
	3	22.17	22.24	0.07	22.00	17.79	-4.21
	4	20.26	20.82	0.56	25.79	22.21	-3.58
	5	23.63	23.57	-0.06	14.01	16.19	2.18
	6	25.62	25.15	-0.47	19.00	19.82	0.82
M2	1	17.71	17.79	0.08	13.49	13.25	-0.24
	2	20.44	20.87	0.42	16.21	16.78	0.57
	3	17.57	17.53	-0.04	12.11	11.39	-0.72
	4	17.04	18.36	1.32	12.48	11.45	-1.04
	6	24.24	25.83	1.58	14.75	11.77	-2.98

Note. [a]Matching variable selection; [b]Unstandardised variance of the spirituality state factor assigned to W17; [c]Unstanardised variance of the spirituality state factor assigned to W20; [d]Difference of the unstandardised variances of the spirituality state factors assigned to the two studied waves (values above *0* represent increases in variance).

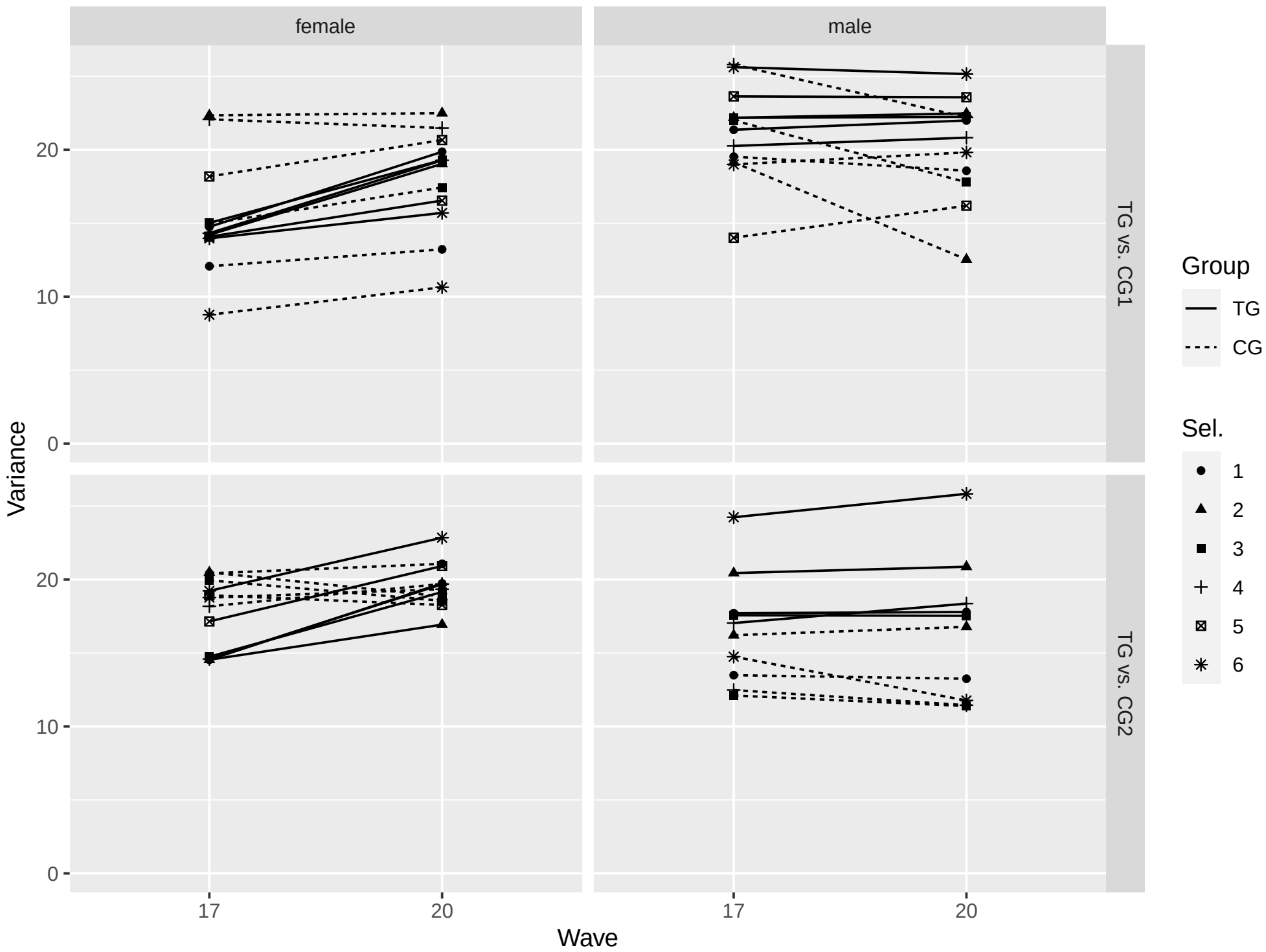

Figure S6: Variances of the spirituality state factors in the latent state models by TG-CG contrast, gender, study group and matching variable selection

Confirming the trend observed in the manifest standard deviations, latent state spirituality variance slightly increased in first-time mothers while it remained stable in most other groups and slightly declined in some childless men.

References

Bandura, A. (1977). Self-efficacy: toward a unifying theory of behavioral change. *Psychological Review, 84*(2), 191-215.

Beck, E. D. & Jackson, J. J. (2022). A mega-analysis of personality prediction: Robustness and boundary conditions. *Journal of Personality and Social Psychology, 122*(3), 523-553.

Berman, Z., Thiel, F., Dishy, G. A., Chan, S. J. & Dekel, S. (2020). Maternal psychological growth following childbirth. *Archives of Women's Mental Health*, 1-8.

Bundesamt für Statistik. (2015). Newsletter Demos. Demografisches Porträt der Regionen [Newsletter Demos. Demographical portrait of the regions]. (BFS number: 349883). https://www.bfs.admin.ch/bfsstatic/dam/assets/349883/master

Bundesamt für Statistik. (2016). Erstgeburten nach Zivilstand der Mutter [Primogeniture by civil status of the mother]. (BFS number gr-d-01.04.01.01.30-su). Retrieved from: https://www.bfs.admin.ch/bfs/de/home/statistiken/kataloge-datenbanken/grafiken.assetdetail.282117.html

Bundesamt für Statistik. (2017). Familien in der Schweiz. Statistischer Bericht 2017. [Families in Switzerland. Statistical report 2017]. (BFS number: 1010-1700). Neuchâtel: BFS.

Bundesamt für Statistik. (2019). Altersspezifische Geburtenziffer nach Staatsangehörigkeit der Mutter, 1986 und 2016 [Age-specific birth rate by nationality of the mother, 1986 and 2016]. (BFS number gr-d-01.04.01.02.01). Retrieved from: https://www.bfs.admin.ch/bfs/de/home/statistiken/kataloge-datenbanken/grafiken.assetdetail.6447837.html

Bundesamt für Statistik. (2020a). Durchschnittsalter der Mutter bei Geburt nach Staatsangehörigkeit [Mean age of the mother at birth by nationality]. (BFS number gr-d-01.04.01.02.03-su). Retrieved from: https://www.bfs.admin.ch/bfs/de/home/statistiken/kataloge-datenbanken/grafiken.assetdetail.14347625.html

Bundesamt für Statistik. (2020b). Lebendgeburten nach Alter der Mutter und des Vaters [Live births by age of the mother and the father]. (BFS number gr-d-01.04.01.01u01.04-su). Retrieved from: https://www.bfs.admin.ch/bfs/de/home/statistiken/kataloge-datenbanken/grafiken.assetdetail.12887400.html

Cobb-Clark, D. A. & Schurer, S. (2012). The stability of big-five personality traits. *Economics Letters, 115*(1), 11-15.

Davis, E. B., Day, J. M., Lindia, P. A. & Lemke, A. W. (2023). Religious/spiritual development and positive psychology: toward an integrative theory. In E. B. Davis, E. L. Worthington Jr. & S. A. Schnitker (Eds.), *Handbook of positive psychology, religion, and spirituality* (pp. 279-299). Cham: Springer.

Eid, M. (2000). A multitrait-multimethod model with minimal assumptions. *Psychometrika, 65*(2), 241-261. https://doi.org/10.1007/BF02294377

Eid, M. & Kutscher, T. (2014). Statistical models for analyzing stability and change in happiness. In K. M. Sheldon & R. E. Lucas (Eds.), *Stability of happiness. Theories and evidence on whether happiness can change* (pp. 261 – 297). Amsterdam: Elsevier.

Eid, M., Gollwitzer, M. & Schmitt, M. (2015). *Statistik und Forschungsmethoden (4., überarb. und erw. Aufl.)* [Statistics and research methods. (4th, revised and extended ed.)]. Weinheim: Beltz.

Greifer, N. (2023). Covariate balance tables and plots: a guide to the cobalt package. https://cran.r-project.org/web/packages/cobalt/vignettes/cobalt.html

Harris, K. A., Howell, D. S. & Spurgeon, D. W. (2018). Faith concepts in psychology: Three 30-year definitional content analyses. *Psychology of Religion and Spirituality, 10*(1), 1-29.

Hill, P. C., Pargament, K. I., Hood, R. W., McCullough, J. M. E., Swyers, J. P., Larson, D. B. et al. (2000). Conceptualizing religion and spirituality: Points of commonality, points of departure. *Journal for the Theory of Social Behaviour, 30*(1), 51-77.

Ho, D. E., Imai, K., King, G., & Stuart, E. A. (2011). MatchIt: Nonparametric preprocessing for parametric causal inference. *Journal of Statistical Software, 42*(8), 1-28. https://www.jstatsoft.org/v42/i08/

Holland, P. W. (1986). Statistics and causal inference. *Journal of the American Statistical Association, 81*(396), 945-960.

Hu, L. T. & Bentler, P. M. (1999). Cutoff criteria for fit indexes in covariance structure analysis: Conventional criteria versus new alternatives. *Structural Equation Modeling: a Multidisciplinary Journal, 6*(1), 1-55.

Huber, S. (2008). Kerndimensionen, Zentralität und Inhalt: Ein interdisziplinäres Modell der Religiosität [Core-dimensions, centrality and content: An interdisciplinary model of religiosity]. *Journal für Psychologie, 16*(3). Retrieved from https://journal-fuer-psychologie.de/article/view/202

Huber, S., Allemand, M. & Huber. O. W. (2011). The relation between forgiveness by God and human forgiveness. The centrality of the religiosity makes the difference. *Archive for the Psychology of Religion, 33*, 115-134.

Huber, S. & Huber, O. W. (2012). The centrality of religiosity scale (CRS). *Religions, 3*(3), 710-724.

ISSP Research Group (1993). *International Social Survey Programme: Religion I - ISSP 1991* (ZA2150 Data file Version 1.0.0). Cologne: GESIS Data Archive. doi: 10.4232/1.2150

ISSP Research Group (2000). *International Social Survey Programme: Religion II - ISSP 1998* (ZA3190 Data file Version 1.0.0). Cologne: GESIS Data Archive. doi: 10.4232/1.3190

ISSP Research Group (2018). *International Social Survey Programme: Religion III - ISSP 2008* (ZA4950 Data file Version 2.3.0). Cologne: GESIS Data Archive. doi: 10.4232/1.13161

ISSP Research Group (2020). *International Social Survey Programme: Religion IV - ISSP 2018* (ZA7570 Data file Version 2.1.0). Cologne: GESIS Data Archive. doi: 10.4232/1.13629

Kenny, D. A., Kaniskan, B. & McCoach, D. B. (2015). The performance of RMSEA in models with small degrees of freedom. *Sociological Methods and Research, 44*(3), 486-507.

Lewis, C. A., Bunting, B. P., Shevlin, M. E. & Joseph, S. (1995). Confirmatory factor analysis of the satisfaction with life scale: Replication and methodological refinement. *Perceptual and Motor Skills, 80*(1), 304-306.

Luhmann, M., Lucas, R. E., Eid, M. & Diener, E. (2013). The prospective effect of life satisfaction on life events. *Social Psychological and Personality Science, 4*(1), 39-45.

Pavot, W. & Diener, E. (2008). The satisfaction with life scale and the emerging construct of life satisfaction. *The Journal of Positive Psychology, 3*(2), 137-152.

Pargament, K. I. (1999). The psychology of religion and spirituality? Yes and no. *The International Journal for the Psychology of Religion, 9*(1), 3-16. https://doi.org/10.1207/s15327582ijpr0901_2

Rantanen, J., Metsäpelto, R. L., Feldt, T., Pulkkinen, L. E. A. & Kokko, K. (2007). Long-term stability in the Big Five personality traits in adulthood. *Scandinavian Journal of Psychology, 48*(6), 511-518.

R Core Team (2022). R: A language and environment for statistical computing (version 4.2.2) [Computer software]. https://www.R-project.org/

Rohrer, J. M. (2018). Thinking clearly about correlations and causation: Graphical causal models for observational data. *Advances in Methods and Practices in Psychological Science, 1*(1), 27-42.

Rosseel, Y. (2012). lavaan: An R package for Structural Equation Modeling. *Journal of Statistical Software, 48*(2), 1-36. http://www.jstatsoft.org/v48/i02/

Rotter, J. B. (1966). Generalized expectancies for internal versus external control of reinforcement. *Psychological Monographs: General and Applied, 80*(1), 1-28.

Rozen, G., Taubman–Ben-Ari, O., Strauss, T. & Morag, I. (2018). Personal growth of mothers of preterms: Objective severity of the event, subjective stress, personal resources, and maternal emotional support. *Journal of Happiness Studies, 19*(7), 2167-2186.

Schermelleh-Engel, K., Moosbrugger, H. & Müller, H. (2003). Evaluating the fit of structural equation models: Tests of significance and descriptive goodness-of-fit measures. *Methods of Psychological Research Online, 8*(2), 23-74.

Schnell, T. (2012). Spirituality with and without religion—Differential relationships with personality. *Archive for the Psychology of Religion, 34*(1), 33-61.

SHP Group (2022). Living in Switzerland Waves 1-22 + Covid 19 data [Dataset]. Lausanne: FORS - Swiss Centre of Expertise in the Social Sciences. Financed by the Swiss National Science Foundation, distributed by FORS. doi: 10.48573/1nav-wy98

Steyer, R., Eid, M. & Schwenkmezger, P. (1997). Modeling true intraindividual change: True change as a latent variable. *Methods of Psychological Research Online, 2*(1), 21-33.

Steyer, R. & Eid, M. (2001). *Messen und Testen* [Measuring and testing] (2nd, corrected ed.). Berlin: Springer.

Stuart, E. A., & Rubin, D. B. (2007). Best practices in quasi-experimental designs. Matching methods for causal inference. In J. Osborne (Ed.), *Best practices in quantitative methods* (pp. 155-176). Sage.

Swiss Federal Office for Spacial Development (2005). Typologies des espaces territoriaux utilisées dans le cadre du monitoring de l'espace rural [Typologies of territorial areas used in the monitoring of the rural space]. Retrieved from: https://www.are.admin.ch/are/fr/home/agglomerations-espaces-ruraux/bases-et-donnees/monitoring-des-espaces-ruraux.html

Taasoobshirazi, G. & Wang, S. (2016). The performance of the SRMR, RMSEA, CFI, and TLI: An examination of sample size, path size, and degrees of freedom. *Journal of Applied Quantitative Methods, 11*(3), 31-39.

United Nations Educational, Scientific and Cultural Organization (2003). International Standard Classification of Education, ISCED 1997. In: J. H. P. Hoffmeyer-Zlotnik, C. Wolf (Eds.), *Advances in cross-national comparison* (pp. 195-220). Boston: Springer. https://doi.org/10.1007/978-1-4419-9186-7_10

Voorpostel, M., Tillmann, R, Lebert, F., Kuhn, U., Lipps, O., Ryser, V.-A. et al. (2022). *Swiss Household Panel Userguide (1999-2020), Wave 22, January 2022.* Lausanne: FORS.

West, S. G., Cham, H., Thoemmes, F., Renneberg, B., Schulze, J. & Weiler, M. (2014). Propensity scores as a basis for equating groups: Basic principles and application in clinical treatment outcome research. *Journal of Consulting and Clinical Psychology, 82*(5), 906.
